# Spread and dynamics of the COVID-19 epidemic in Italy: Effects of emergency containment measures

**DOI:** 10.1073/pnas.2004978117

**Published:** 2020-04-23

**Authors:** Marino Gatto, Enrico Bertuzzo, Lorenzo Mari, Stefano Miccoli, Luca Carraro, Renato Casagrandi, Andrea Rinaldo

**Affiliations:** ^a^Dipartimento di Elettronica, Informazione e Bioingegneria, Politecnico di Milano, 20133 Milano, Italy;; ^b^Dipartimento di Scienze Ambientali, Informatica e Statistica, Università Ca’ Foscari Venezia, 30172 Venezia-Mestre, Italy;; ^c^Science of Complexity Research Unit, European Centre for Living Technology, 30123 Venice, Italy;; ^d^Dipartimento di Meccanica, Politecnico di Milano, 20133 Milano, Italy;; ^e^Department of Aquatic Ecology, Swiss Federal Institute of Aquatic Science and Technology, 8600 Dübendorf, Switzerland;; ^f^Department of Evolutionary Biology and Environmental Studies, University of Zurich, 8057 Zurich, Switzerland;; ^g^Laboratory of Ecohydrology, École Polytechnique Fédérale de Lausanne, 1015 Lausanne, Switzerland;; ^h^Dipartimento di Ingegneria Civile, Edile e Ambientale, Università di Padova, 35131 Padova, Italy

**Keywords:** SARS-CoV-2, spatially explicit epidemiology, disease outbreak scenarios, SEIR models, social contact restrictions

## Abstract

The spread of coronavirus disease 2019 (COVID-19) in Italy prompted drastic measures for transmission containment. We examine the effects of these interventions, based on modeling of the unfolding epidemic. We test modeling options of the spatially explicit type, suggested by the wave of infections spreading from the initial foci to the rest of Italy. We estimate parameters of a metacommunity Susceptible–Exposed–Infected–Recovered (SEIR)-like transmission model that includes a network of 107 provinces connected by mobility at high resolution, and the critical contribution of presymptomatic and asymptomatic transmission. We estimate a generalized reproduction number ($R_{0}$ = 3.60 [3.49 to 3.84]), the spectral radius of a suitable next-generation matrix that measures the potential spread in the absence of containment interventions. The model includes the implementation of progressive restrictions after the first case confirmed in Italy (February 21, 2020) and runs until March 25, 2020. We account for uncertainty in epidemiological reporting, and time dependence of human mobility matrices and awareness-dependent exposure probabilities. We draw scenarios of different containment measures and their impact. Results suggest that the sequence of restrictions posed to mobility and human-to-human interactions have reduced transmission by 45% (42 to 49%). Averted hospitalizations are measured by running scenarios obtained by selectively relaxing the imposed restrictions and total about 200,000 individuals (as of March 25, 2020). Although a number of assumptions need to be reexamined, like age structure in social mixing patterns and in the distribution of mobility, hospitalization, and fatality, we conclude that verifiable evidence exists to support the planning of emergency measures.

## Significance

The ongoing pandemic of COVID-19 challenges globalized societies. Scientific and technological cross-fertilization yields broad availability of georeferenced epidemiological data and of modeling tools that aid decisions on emergency management. To this end, spatially explicit models of the COVID-19 epidemic that include e.g. regional individual mobilities, the progression of social distancing, and local capacity of medical infrastructure provide significant information. Data-tailored spatial resolutions that model the disease spread geography can include details of interventions at the proper geographical scale. Based on them, it is possible to quantify the effect of local containment measures (like diachronic spatial maps of averted hospitalizations) and the assessment of the spatial and temporal planning of the needs of emergency measures and medical infrastructure as a major contingency planning aid.

**Fig. 1.**
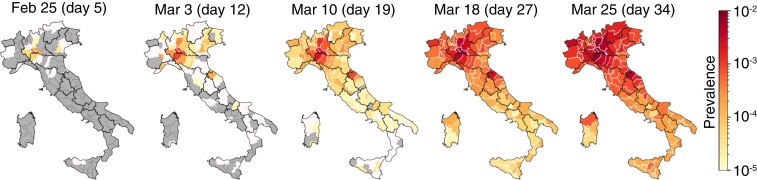
Evolution of the ratio of confirmed cases/resident population in Italy. The spatial spread over time of COVID-19 is plotted from February 25 to March 25, 2020. See also animations from day 5 to day 34 in Movies S1 and S2.

**Fig. 2.**
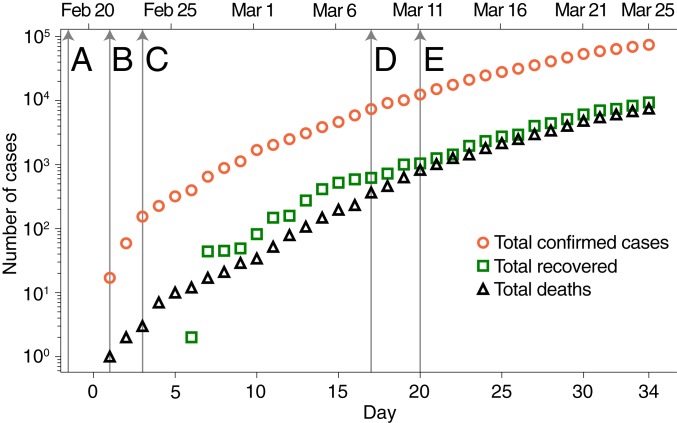
Time evolution of the COVID-19 epidemic in Italy. Time marks are as follows: *a*, the first patient with suspected local transmission is hospitalized in Codogno; *b*, first confirmed cases; and *c*, *d*, and *e*, main containment measures enforced by the Italian government (detailed in *Materials and Methods*).

Since December 2019, a cluster of pneumonia cases in the city of Wuhan, China (1–7), has developed into a pandemic wave currently ravaging several countries (8–12). The pathogen causing the acute pneumonia among affected individuals is the new coronavirus severe acute respiratory syndrome coronavirus 2 (SARS-CoV-2) (8, 9, 13, 14). As of March 25, 2020, a total of 467,593 cases of coronavirus disease 2019 (COVID-19) have been confirmed worldwide in 181 countries (15). In Italy, a hotspot of the pandemic, the count, as of March 25, 2020, refers to 74,386 total confirmed cases and 7,503 deaths (15–18) (Figs. 1 and 2). The well-monitored progress of the wave of infections highlighted in Fig. 1 (for complete documentation, see *SI Appendix* and Movies S1 and S2) clearly speaks of decisive spatial effects. Models are often used to infer key processes or evaluate strategies for mitigating influenza/SARS pandemics (5, 6, 12, 19–24). Early attempts to model the spread of COVID-19 in Italy (25, 26) aired concern regarding the Italian national health system’s capacity to respond to the needs of patients (27), even considering aggregate isolation measures. However, modeling predictions therein disregard the observed spatial nature of the progress of the wave of infections, and can treat only indirectly the effects of containment measures. Critically, therefore, to deal with what could happen next in terms of forthcoming policy decisions, one needs to deal with spatially explicit models (12, 28, 29).

We model in space and time the countrywide spread of the COVID-19 epidemic in Italy (*Materials and Methods*), for which detailed epidemiological data are continuously updated and made public (16, 18, 30). Data are only a proxy of the actual epidemiological conditions because 1) the number of infected people on record depends on the sampling effort, namely, the number of specimen collections (swabs) from persons under investigation (PUIs) (implications discussed in *Materials and Methods*, and *SI Appendix*); and 2) the effects of systematic errors or bias in the official data result mainly in underreporting and need to be considered. In fact, underreporting may apply even to fatality counts, yet to a lesser extent with respect to reported infections. Hospitalizations are known, but may underestimate the actual situation because cases with mild symptoms (termed asymptomatics in the model) are not hospitalized, for example, due to saturation of the carrying capacity of the sanitary structures. For these reasons, we believe that these major sources of uncertainty could be partially offset by estimating the model parameters by using only reported data on hospitalizations, fatality rates, and recovered individuals, without considering the statistics on reported infections.

We concentrate on estimating the effects of severe progressive restrictions posed to human mobility and human-to-human contacts in Italy (*Materials and Methods*; see also timeline in Fig. 2).

Our quantitative tools (31–36) are Markov chain Monte Carlo (MCMC) parameter estimation (*Materials and Methods*) and the extended use of a metacommunity Susceptible–Exposed–Infected–Recovered (SEIR)-like disease transmission model (*Materials and Methods*) that includes a network of 107 nodes representative of closely monitored Italian provinces and metropolitan areas (second administrative level). We use all publicly available epidemiological data, detailed information about human mobility among the nodes (i.e., fluxes and connections; *Materials and Methods*), and updates on containment measures and their effects by relying also on mobile phone tracking (37). Their effective implementation is generally a matter of concern (38). As explained in *Materials and Methods*, the compartments of the model are susceptibles (S), exposed (E), presymptom (P), symptomatic infectious (I), and asymptomatic infectious (A) (core SEPIA model) (*Materials and Methods*). The results of parameter estimation allow us to analyze the relative importance of containment measures and of the various epidemiological compartments and their process parameters, which were also discussed in the context of spatially implicit models, for example, in refs. 3–6, 13, 14, 25, 26, and 39. This is true, in particular, for the critical compartments of asymptomatic (5, 6, 9, 28) and of presymptom infectious individuals (see below). As the model is spatially explicit, we implement a generalized reproduction number, that is, the spectral radius of a next-generation matrix (NGM) (35, 36, 40, 41), that measures the potential spread in the absence of containment interventions (*Materials and Methods*). We also calculate the dominant eigenvalue (and the corresponding eigenvector) of a suitable Jacobian matrix that provides an estimate of the exponential rate of case increase within a disease-free population, and the related asymptotic geographic distribution of the infectious (35, 36). In case of time-varying parameters, significant technical complications would arise [e.g., computing Floquet (42) or Lyapunov exponents (43)]. Numerical simulation then supplies directly the desired scenarios in the presence of time-varying containment measures.

A critical issue concerns the description of human mobility that determines exposures and thus, ultimately, the extent of the contagion (28). Although the dense social contact networks characteristic of urban areas may be seen as the fabric for disease propagation, calling for specific treatment of “synthetic populations” (44, 45), here, because of 1) the large number of cases involved, 2) the countrywide scale of the domain, and 3) the scope of the study aimed at broad large-scale effects of emergency management, we choose to represent node-to-node fluxes from data neglecting demographic stochasticity (but see refs. 14 and 29) and social contact details. Stochasticity is considered through locally estimated seeding of cases surrogating randomness in mobility, which had been considered earlier in the framework of branching processes (14). Coupling this information with the epidemiological data allows us to estimate the effects of enforced or hypothesized containment measures in terms of averted hospitalizations. This yields scenarios on what course the disease might have taken if different measures had been implemented.

## Results

**Fig. 3.**
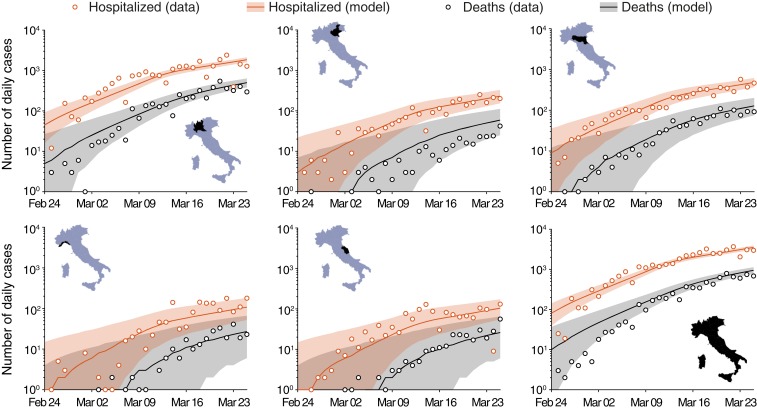
Reported and simulated aggregate number of new daily hospitalized cases and deaths for COVID-19 spread in Italy (February 24 to March 25, 2020) (16, 17, 18). Computed results are obtained for the set of parameters shown in Table 2. Lines represent median model results, while shaded areas identify 95% CIs. Clockwise from lower right corner (see *Insets*): Italy, Marche, Liguria, Lombardia, Veneto, and Emilia-Romagna. Other regions are shown in *SI Appendix*, Fig. S12.

$R_{0}=3.60$ (95% CI: 3.49 to 3.84) is the estimate of the initial generalized reproduction number, which includes mobility and the spatial distribution of communities (*Materials and Methods*). The full set of estimated parameters is reported in Table 2, while the comparisons between model simulations and data are shown in Fig. 3 for five representative regions and the whole of Italy (the remaining regions are reported in *SI Appendix*, Fig. S12). An animation showing the comparison between the simulated and reported spatiotemporal evolution of the outbreak is reported as Movie S2.

As noted in *Materials and Methods*, a spatially explicit generation matrix $K_{L}$ describes the contributions of presymptom infectious, infectious people with severe symptoms, and infectious people with no/mild symptoms to the production of new infections close to the disease-free equilibrium. A graph representation of the spatial NGM (*Materials and Methods*) is shown later (see Fig. 5*C*). Crucially, the dominant eigenvalue ($g_{0}=0.24\;d^{-1}$ [95% CI: 0.22 to 0.26]) of the system’s Jacobian matrix, evaluated at the disease-free equilibrium, provides an estimate of the initial exponential rate of case increase. The eigenvector corresponding to the leading eigenvalue, which represents the expected spatial distribution of cases in the asymptotic phase of exponential epidemic growth (35, 36), is shown in *SI Appendix*, Fig. S13. The main result emerging therein is that a completely uncontrolled epidemic would have eventually hit mostly the main metropolitan areas.

We estimate that containment measures and changes in social behavior and awareness have progressively reduced the transmission by 45% (95% CI: 42 to 49%). The first set of measures resulted in a reduction of the transmission parameter, $\beta_{P}$ in Table 2, by 18%, while the second set of measures further reduces it by an additional 34%.

**Fig. 4.**
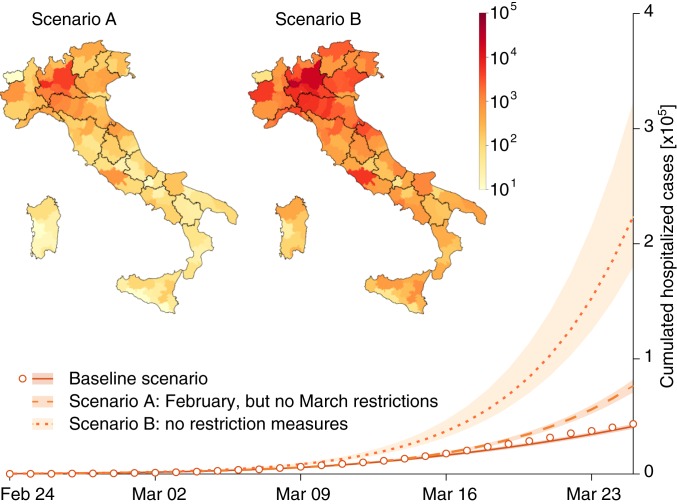
Hospitalizations (graph) and increases of hospitalization demands (maps), based on scenarios of modified transmission of COVID-19 in Italy. Data (white circles) and the lower curve (baseline scenario) show, respectively, observations and model projections of the cumulative hospitalizations as a result of the actual disease spread constrained by the enforcement of the scheduled restrictions of the Italian government (see arrows in Fig. 2). The middle curve (dashed line, scenario A) represents the expected demand of hospitalizations, had the government not imposed the further March restrictions. The map of scenario A shows the corresponding expected increase of hospitalization demand with respect to the baseline as of March 25, 2020. The uppermost curve (dotted line, scenario B) shows the expected hospitalizations, had no restrictive measure been imposed. The map of scenario B shows the corresponding increase of hospitalization demand.

Fig. 4 reports, for the whole of Italy, three different scenarios in terms of the cumulative number of hospitalizations. We chose to represent only this state variable for clarity, and for the obvious implications on emergency management. The baseline shown in Fig. 4 is the one in which the model has been identified (lower curve and data) by including changes in the spatial human mobility and in collective social behavior, jointly with their timing (*Materials and Methods*). The other two curves represent “what if” scenarios. The first (scenario A), corresponding to the middle curve in the graph, is the one in which only the first set of containment measures is implemented. The second (scenario B), portrayed by the upper curve, is obtained by excluding all containment measures. The comparison between scenarios allows us to estimate the number of averted cases (excess of hospitalization demand with respect to the baseline), jointly with their spatial distributions (maps of scenarios A and B in Fig. 4). The actual number of averted cases is obtained by the difference of hospitalizations between the baseline and scenario B (no containment measures). We obtain a median of $0.226\cdot 10^{6}$ averted cases (95% CI: $0.172\cdot 10^{6}\;\text{to}\;0.347\cdot 10^{6}$), as of March 25, 2020.

**Fig. 5.**
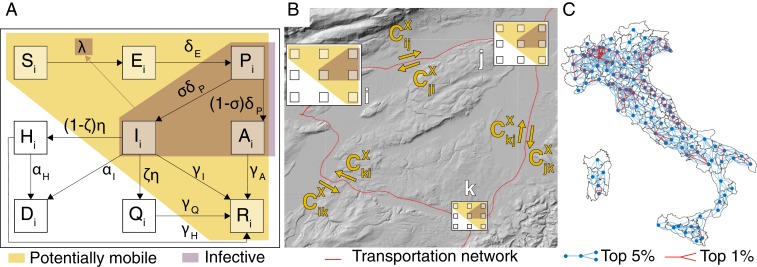
Schematic representation of the spatially explicit epidemiological model. (*A*) Local transmission dynamics (as in Eq. **1**). (*B*) Connections between the local communities. (*C*) Main routes of COVID-19 propagation in Italy as estimated via NGM (*SI Appendix*).

An analogous plot for the total averted infections is shown in *SI Appendix*, Fig. S14. Therein, one notes that the total infections are calculated by integrating in time the force of the infection, that is, the sum over all 107 nodes i of the flux ($\lambda_{i}S_{i}\left(t\right)$; see *Materials and Methods*) leaving the susceptibles compartment. The number of averted cases is computed as discussed for the results on hospitalizations in Fig. 4. The median number of averted infections due to the implementation of all restriction measures is $6.49\cdot 10^{6}$ (95% CI: $4.81-10.1\cdot 10^{6}$). Our median estimate of the total number of infections, as of March 25, 2020, is approximately 733,000 individuals.

## Discussion

Globalized societies are challenged by emerging diseases, in many cases, zoonoses (46), often related to climate change (47, 48). COVID-19 is a paradigmatic example of zoonosis whose pandemic character is tied to the globalized travel that spread the contagion in a few months (11, 12). Scientific and technological advances in a variety of fields provide a broad availability of data and modeling tools that must inform decision-making on emergency management. This exercise intends to contribute to this cross-fertilization.

Here, we have developed and implemented a spatial framework for the ongoing COVID-19 emergency in Italy, which is characterized by evident spatial signatures (SI Movies S1 and S2 clearly show the radiation of the epidemic along highways and transportation infrastructures). Our analysis of the contributions of different compartments points to the important role played by presymptom infectious in the disease spread and growth (Table 2). The estimated high presymptomatic transmission parameter $\beta_{P}$, with respect to the transmission rates of symptomatic and asymptomatic infectious $\beta_{I,A}$, reproduces field epidemiological evidence (49) and provides support for explicitly accounting for the presymptomatic compartment in the SEPIA model. This result may have profound implications for containment measures [possibly even centralized quarantines (50)], because it may suggest the need for a massive swab testing to identify and isolate presymptomatic infectious cases (51). This underpins that greatly improved contact tracing has the potential to stop the spread of the epidemic if reliably used on sufficiently large numbers (52).

The lockdown introduced in Italy by the second set of measures was far more stringent than the first. As a consequence, noted in *Results*, the transmission rates have been progressively and significantly reduced. The different age of the measures (current time minus its onset) has therefore produced different effects. This needs to be accounted for, to properly judge their effectiveness. At first sight, in fact, the effects of the second set of measures taken in March could erroneously appear less important than in reality (A in Fig. 4). Obviously, the effects of the second set of measures will fully display their importance after March 25, 2020, the end date for our analysis.

Our study presents a number of simplifications and limitations that, however, do not impair our main conclusions. Specifically, 1) although the human effort involved in the collection of epidemiological data has been major, the granularity of available data is limited in time, spatial resolution, and individual information [for instance, the only published assessment of mobility changes in Italy following lockdown (37) refers to publicly unavailable data; properly anonymized call detail records have been useful in other epidemic and endemic contexts (34, 53, 54)]; 2) should anonymized individual information from hospitals and laboratories be available, a proper probability distribution of relevant rates and periods (e.g., latency, incubation, infection) could be employed by any modeling approaches (see ref. 55 for estimates based on high data granularity regarding the Lombardy region); and 3) the effect of age structure (56) in terms of differential mobility, social contact patterns, vulnerability, and case fatality ratio [often associated with hyperinflammation in elderly people (57)] would need to be included, therefore relying on higher granularity of data (39). Further developments may also deal with operational predictions based on our modeling framework, once coupled, for example, to ensemble Kalman filtering and updates of parameter estimates and state variables, as already customary in other epidemiological studies (58–60), and currently employed only in a few studies on COVID-19 (28, 61). The spatial nature of the model, in fact, would possibly aid the planning of the agenda for differential mobility restrictions and deployments of local medical supplies and staff tuned to local epidemiological and logistic conditions. We do not attempt, at this stage, to simulate the long-term evolution of the disease dynamics, because it depends on the time evolution of the conditions determining critical epidemiological parameters such as people’s behavior and contact rates, further restrictions to mobility, or the discovery of new specific antiviral drugs (62).

We propose an estimate of total infections computed from our model (*SI Appendix*, Fig. S14). We find a significantly larger figure than in the official counts: as of March 25, 2020, we estimate a median of about 600,000 contagions, whereas the official count of confirmed infections is 74,386. This result does not confirm earlier, much larger estimates (63). However, the estimation of certain key epidemiological parameters proves remarkably similar in ref. 63 and in this paper, possibly providing an avenue for future convergence.

We conclude that a detailed spatially explicit model of the unfolding COVID-19 spread in Italy, inclusive of the imposed restriction measures, closely reproduces the empirical evidence. This allows us to draw significant indications of the key processes involved in the contagion, together with their time-dependent nature and parameters. When applied by restarting the simulation while removing the restrictive measures, the model shows, unequivocally, that their effects have been decisive. Indeed, the total expected number of averted hospitalizations in Italy, a significant measure of the needs of emergency management (and the less error-prone epidemiological measure), ran on the order of 200,000 cases up to March 25, 2020, for the whole country, and is known with sufficient spatial granularity. Implications on fatality rates and emergency management are direct, as the capacity of the Italian medical facilities—although continuously expanding—is known at each relevant time. Thus our results bear social and economic significance, because they unquestionably support drastic governmental decisions.

## Materials and Methods

### Epidemiological Model.

Many models have been developed to describe the course of the COVID-19 pandemic in individual countries or at the global scale. Actually, no clear consensus has been reached on the different compartments that should be included in a proper model. Our model choice was motivated by a review of the existing approaches. Most models assume a standard SEIR structure but make different hypotheses on the nature of the different compartments and their respective residence times. Some of the key epidemiological features characteristic of COVID-19 are summarized in Table 1, together with the appropriate references, while the different approaches are described in more detail in *SI Appendix*.

**Table 1.** Key epidemiological periods to model the dynamics of COVID-19 together with values of $R_{0}$

Period	Values (days)	Reference
Latency	7	(5, 10)
	5.2 (${CI}_{95\%}$ = [4.1–7.0])	(4, 9, 14)
	3.44–3.69	(28)
Serial interval	7.5 (mean, ${CI}_{95\%}$ = [5.5–19], n = 6	(64)
	5.1 (mean, ${CI}_{95\%}$ = [1.3–11.6], n = 8579)	(65)
	4.56 (mean, ${CI}_{95\%}$ = [2.69–6.42], n = 93)	(66)
	4.22 (mean, ${CI}_{95\%}$ = [3.43–5.01], n = 135)	
	4.4 (mean, ${CI}_{95\%}$ = [2.9–6.7], n = 21)	(67)
	4.0 (mean, ${CI}_{95\%}$ = [3.1–4.9], n = 28)	(68)
	3.96 (mean, ${CI}_{95\%}$ = [3.53–4.39], n = 468)	(49)
Incubation	9 (mean, ${CI}_{95\%}$ = [7.92–10.2], n = 135)	(66)
	7.1 (mean, ${CI}_{95\%}$ = [6.13–8.25], n = 93)	
	6.6 (mean, ${CI}_{95\%}$ = [0.7–19.0], n=90)	(55)
	5.1 (median, ${CI}_{95\%}$ = [4.5–5.8]	(69)
	5.2 (mean, ${CI}_{95\%}$ = [4.1–7.0], n = 10)	(9)
	6.4 (mean, ${CI}_{95\%}$ = [5.6–7.7], n = 88)	(70)
	5 (mean, ${CI}_{95\%}$ = [4.2–6.0], n = 52)	(71)
	5.6 (mean, ${CI}_{95\%}$ = [5.0–6.3], n = 158)	
	5.2 (mean, ${CI}_{95\%}$ = [1.8–12.4], N = 8579)	(65)
	4.8 (mean, SD = 2.6, n = 830)	(64)
	≅ latency	(12–14)
	lag of 5	(4)
Infectious	2.16 (range 1.64–3.10)	(5)
	2.4	(13)
	2.9	(14)
	3.5	(28)
	2–8	(12)
$R_{0}$	2.2 (${CI}_{95\%}$ = [1.4–3.9])	(9)
	2.6 (CI 2.1−5.1)	(72)
	3.1 (${CI}_{95\%}$ = [2.9–3.2])	(55)
	4.5 (${CI}_{95\%}$ = [4.4–4.6])	(73)
	4.4 (${CI}_{95\%}$ = [4.4–4.6])	(73)
	6.47 (${CI}_{95\%}$ = [5.71–7.23])	(5)

Here, we propose and use a model that is elaborated moving from the basic local scheme of ref. 5. By introducing the new compartment of presymptomatic infectious individuals, we account for a peculiar epidemiological state of the disease under study. Empirical evidence (see again Table 1) shows, in fact, that the serial interval of COVID-19 tends to be shorter than the incubation period, thus suggesting that a substantial proportion of secondary transmission can occur prior to illness onset (68). Presymptom transmission appears to play an important role in speeding up the spread of the disease within a community, accounting for around 12.6% of case reports in China (49), 48% in Singapore, and 62% in Tianjin, China (74). The core of our model is thus termed SEPIA and includes the following compartments: Susceptible (S), Exposed (E), Presymptomatic (P), Infected with heavy symptoms (I), Asymptomatic/mildly symptomatic (A), Hospitalized (H), Quarantined at home (Q), Recovered (R), and Dead (D) individuals.

The local dynamics of transmission is given by

$$\begin{array}{cc} \dot{S} & =-\lambda S\\ Ė & =\lambda S-\delta_{E}E\\ \dot{P} & =\delta_{E}E-\delta_{P}P\\ İ & =\sigma \delta_{P}P-\left(\eta +\gamma_{I}+\alpha_{I}\right)I\\ Ȧ & =\left(1-\sigma \right)\delta_{P}P-\gamma_{A}A\\ \dot{H} & =\left(1-\zeta \right)\eta I-\left(\gamma_{H}+\alpha_{H}\right)H\\ \dot{Q} & =\zeta \eta I-\gamma_{Q}Q\\ \dot{R} & =\gamma_{I}I+\gamma_{A}A+\gamma_{H}H\\ \dot{D} & =\alpha_{I}I+\alpha_{H}H. \end{array}$$ [1]

In the model, susceptible individuals (S) become exposed to the viral agent upon contact with infectious individuals, assumed to be those in the presymptomatic, heavily symptomatic, or asymptomatic/mildly symptomatic classes. Although the hypothesis might not hold for some very sparse communities, we assume frequency-dependent contact rates (as most authors do), so that exposure occurs at a rate described by the force of infection,

$$\lambda =\frac{\beta_{P}P+\beta_{I}I+\beta_{A}A}{S+E+P+I+A+R},$$

where $\beta_{P}$, $\beta_{I}$, and $\beta_{A}$ are the specific transmission rates of the three infectious classes. Exposed individuals (E) are latently infected, that is, still not contagious, until they enter the presymptom stage (at rate $\delta_{E}$) and only then become infectious. Presymptomatic individuals (P) progress (at rate $\delta_{P}$) to become symptomatic infectious individuals who develop severe symptoms (with probability σ). Alternatively, they become asymptomatic/mildly symptomatic individuals (with probability 1−σ). Symptomatic infectious individuals (I) exit their compartment if/when 1) they are isolated from the community (at rate η) because a fraction 1−ζ of them is hospitalized, while a fraction ζ is quarantined at home, 2) they recover from infection (at rate $\gamma_{I}$), or 3) they die (at rate $\alpha_{I}$). Asymptomatic/mildly symptomatic individuals (A), on the other hand, leave their compartment after having recovered from infection (at rate $\gamma_{A}$). Hospitalized individuals (H) may either recover from infection (at rate $\gamma_{H}$) or die because of it (at rate $\alpha_{H}$), while home-isolated individuals (Q) leave their compartment upon recovery (at rate $\gamma_{Q}$). People who recover from infection or die because of COVID-19 populate the class of recovered (R) and dead (D) individuals, respectively, independently of their epidemiological compartment of origin.

The model is made spatial by coupling n human communities at the suitable resolution via a community-dependent force of infection. It results from local and imported infections due to contacts within the local community or associated with citizens’ mobility. More precisely, the force of infection for community i is given by

$$\lambda_{i}=\overset{n}{\underset{j=1}{\sum }}C_{ij}^{S}\frac{\sum_{Y\in \left\{P,I,A\right\}}\sum_{k=1}^{n}\beta_{Y}C_{kj}^{Y}Y_{k}}{\sum_{X\in \left\{S,E,P,I,A,R\right\}}\sum_{k=1}^{n}C_{kj}^{X}X_{k}},$$

where $C_{ij}^{X}$ (with X∈{S,E,P,I,A,R}) is the probability ($\sum_{j=1}^{n}C_{ij}^{X}=1$ for all i and X) that individuals in epidemiological state X who are from community i enter into contact with individuals who are present at community j as either residents or because they are traveling there from community k (note that i, j, and k may coincide). Details are provided in *SI Appendix*.

A frequently used indicator is the basic reproduction number, namely, the number $R_{0}$ of secondary infections produced by one primary infection in a fully susceptible population. This simple concept works fine in a spatially isolated community, where everything is well mixed at any instant. Instead, if the model parameters are inhomogeneous both in space and in time, the number of secondary infections produced by one primary infection might vary accordingly. Also, $R_{0}$ may depend on people’s behavior and on the control measures being enforced. When a realistic spatial model is introduced to describe the spread in a country, it is necessary to resort to the definition of generalized reproduction numbers based on the spectral radius of a suitable epidemiological matrix (35, 36, 40).

If we consider the spatial model described above in the case when no emergency measures are enforced and people’s behavior does not change, then the basic reproduction number can be calculated as (see *SI Appendix* for the detailed derivation)

$$R_{0}=\rho \left(K_{L}\right)=\rho \left(G_{P}+G_{I}+G_{A}\right),$$

where $\rho \left(K_{L}\right)$ is the spectral radius of the NGM (40) and

$$G_{P}=\frac{\beta_{P}}{\delta_{P}}GC_{P}^{T}\;,\;G_{I}=\frac{\beta_{I}GC_{I}^{T}}{\eta +\gamma_{I}+\alpha_{I}}\;,\;G_{A}=\frac{\beta_{A}}{\gamma_{A}}GC_{A}^{T}$$

are three spatially explicit generation matrices describing the contributions of 1) presymptom infectious, 2) infectious with severe symptoms, and 3) infectious with no/mild symptoms, to the production of new infections close to the disease-free equilibrium. The matrices $C_{X}=\left[C_{ij}^{X}\right]$ (X∈{S,P,I,A}) are row stochastic (i.e., their rows sum up to one) and represent spatially explicit contact probabilities. Matrix $G=NC_{S}\Delta^{-1}$ is constructed as follows: N is a diagonal matrix whose nonzero elements are the population sizes $N_{i}$ of the n communities, $C_{S}$ is the contact matrix for susceptibles, and $\Delta =diag\left(uNC_{S}\right)$, with u being a unitary row vector of size n. Matrix $K_{L}$ is a spatially explicit NGM, whose spatial structure describes the main routes of spatial propagation of the epidemic. Also, the dominant eigenvalue (and the corresponding eigenvector) of the system Jacobian matrix, evaluated at the disease-free equilibrium, provides an estimate of the initial exponential rate of case increase, and the related asymptotic geographic distribution of the infectious (35, 36).

## Data

### Available Data and the Course of the Epidemic.

Here, we use the data released every day at 6 PM (UTC +1 h) by the Dipartimento della Protezione Civile and archived on GitHub (75). At times, data may be just a proxy of the actual state variables. In particular, the number of infected people (be they exposed, presymptomatic, symptomatic, or asymptomatic) depends on the effort being devoted to finding new positive cases, namely, the number of specimen collections (swabs) from PUIs. The standard methodology employed by the Istituto Superiore di Sanit (ISS) for confirming a suspected case is the one used by the European Centre for Disease Prevention and Control (76). According to the bulletin of the ISS (17), a median time between the beginning of symptoms and the confirmed diagnosis (positive swabs) ranges between 3 d and 4 d. Sometimes, however, people test positive even without displaying symptoms (e.g., they are tested because they were in contact with symptomatic infectious). Therefore, it seems that the number of positive swabs may not provide a reliable indication of the number of exposed, and probably little indication of the number of presymptom individuals. Actually, these data seem to provide an idea about the number of people who are infectious and have developed mild symptoms (isolated at home) or more serious symptoms (hospitalized), but much less about those with very mild symptoms who are not always subjected to a test.

### Measures for Mobility Restrictions and Contact Reduction.

The detailed sequence of progressive restrictions posed to human mobility and human-to-human contacts in Italy may be summarized as follows:

A) On February 18, 2020, a patient (dubbed “patient one” by Italian media outlets) is admitted to the emergency room in Codogno (Lombardy, province of Lodi) for pneumonia.
B) On February 21, 2020 (day 1), “patient one” is officially confirmed as a case of COVID-19 by Ospedale Sacco in Milano; local authorities struggle to trace the transmission path, and mass testing of population in the Codogno area starts; by the end of the day other 16 cases in Lombardy are confirmed. A further two cases are confirmed in Veneto.
C) On February 23, 2020 (day 3), as no clear link to travelers from China emerges, evidence for local transmission for “patient one” increases. A second cluster of infections is discovered in Vo’ (Veneto, province of Padua). Ten municipalities in Lombardy and one in Veneto, identified as infection foci, are put under strict lockdown (red areas); some restrictions are enacted in Lombardy, Emilia-Romagna, Veneto, Friuli-Venezia Giulia, Piedmont, and Autonomous Province of Trento.
D) On March 8, 2020 (day 17), the whole of Lombardy and 15 northern Italy provinces are under lockdown. The rest of Italy implements social distancing measures. A leak of a draft of the law implementing these measures prompts a panic reaction, with people leaving northern Italy and moving toward other regions.
E) On March 11, 2020 (day 20), the lockdown area is extended; severe limitations to mobility for the whole nation are instituted.

### Model Implementation and Parameter Estimation.

The model has been implemented at the scale of the second administrative level (mainly provinces and metropolitan areas), which comprises 107 units. Therefore, census mobility fluxes available at the municipal level (7,904 entities) were upscaled to the provincial level (*SI Appendix*). Matrices $C_{X}=\left[C_{ij}^{X}\right]$ (X∈{S,E,P,I,A}) are derived from the mobility data.

We explicitly reproduce in our simulations the effects of the restriction measures described above by 1) restricting access and exit from the red areas (*SI Appendix*, Figs. S5–S7), starting from February 23, 2020, and 2) reducing the fraction of people traveling outside the resident province according to data collected through mobile applications and presented in ref. 37. To simulate the change in social behavior and the increase in social distancing, we assume that the transmission parameters $\beta_{P}$, $\beta_{I}$, and $\beta_{A}$ had a sharp decrease (within 2 d) after the measures announced on February 24 and March 8, 2020, and we estimate those step reductions (Table 2). It should be noted that the reduction in the transmission parameters is due not only to the implementation of restriction measures (e.g., school and office closures) but also to the increased awareness of the population, especially after the first cases were reported.

**Table 2.** List of estimated parameters, MCMC estimates and relevant priors of each parameter with N(a,b) being a normal distribution of average a and SD b, and U(a,b) being a uniform distribution in the interval [*a*,*b*]

Parameter	Median (95% CIs)	Prior
$R_{0}$ (-)	3.60 [3.49, 3.84]	N(2.5,0.25)
$1/\delta_{E}$ (d)	3.32 [3.03, 3.66]	N(4,0.4)
$1/\delta_{P}$ (d)	0.75 [0.61, 1.02]	N(1,0.1)
1/η (d)	4.05 [3.85, 4.29]	N(4,0.4)
$1/\gamma_{I}$ (d)	14.32 [13.64, 15.81]	U(0,100)
$1/\alpha_{I}$ (d)	24.23 [22.35, 26.87]	U(0,100)
$\beta_{A}$/$\beta_{P}$ (-)	0.033 [0.027, 0.0036]	U(0,0.5)
$\beta_{I}$/$\beta_{A}$ (-)	1.03 [0.79, 1.38]	N(1,0.2)
$\beta_{P_{1}}/\beta_{P}$ (-)	0.82 [0.77, 0.86]	U(0,1)
$\beta_{P_{2}}/\beta_{P_{1}}$ (-)	0.66 [0.64, 0.70]	U(0,1)
$\Delta t_{0}$ (d)	34.94 [31.62, 39.30]	U(0,100)
ω (-)	7.84 [7.10, 8.34]	U(0,100)

Posterior distributions are shown in *SI Appendix*, Fig. S15.

Model parameters are estimated in a Bayesian framework by sampling the posterior parameter distribution via the ${DREAM}_{zs}$ (77) implementation of the MCMC algorithm. As testing effort and quarantine policy vary across different Italian regions, we prefer to focus on more reliable variables like the number of hospitalized people, deaths, and patients discharged from the hospital. Specifically, we define the likelihood based on daily numbers of hospitalized cases (flux ηI), discharged from hospital ($\gamma_{H}H$), and recorded deaths ($\alpha_{H}H$) at the province level. To account for possible overdispersion of the data, we assume that each data point follows a negative binomial distribution (78, 79) with mean μ, equal to the value predicted by the model, and variance equal to ωμ (NB1 parametrization). We estimated the parameter ω.

To account for the temporal evolution of the epidemics prior to the first detected patient, we impose an initial condition of one exposed individual in the province of Lodi (where the first cases emerged) $\Delta t_{0}$ days before February 24, 2020, and we estimate this parameter. During this period, the disease was likely seeded into other provinces via either human mobility or importation of cases from abroad. The process during this period was likely characterized by high demographic stochasticity due to the low number of involved individuals, and thus it can hardly be captured by our deterministic modeling of average mobility and disease transmission. Moreover, long-distance travels and importation of cases are not accounted for in the data used to represent human mobility, which mostly reflect commuting fluxes for work and study purposes. Therefore, to include this possible seeding effect, we estimated also the initial condition in each province. Specifically, this is done by seeding a small fraction of exposed individuals at the beginning of the simulation.

The list of estimated parameters is reported in Table 2. The parameter $\beta_{P}$ is expressed as a function of the local reproduction number $R_{0}$ (*SI Appendix*). $\beta_{P_{1}}$ and $\beta_{P_{2}}$ represent the values of the parameter $\beta_{P}$ after the measures introduced on February 22 and on March 8, 2020, respectively. The fraction of symptomatic infected being quarantined, ζ, is assumed to be equal to 0.4, that is, the average value for Italy during the observed period (17). During preliminary tests, we found a correlation between the asymptomatic fraction (1−σ) and the asymptomatic transmission rate $\beta_{A}$. Indeed, in the early phase of an epidemic, when the depletion of susceptible is not significant, it is difficult to estimate the role or asymptomatics. We therefore fixed σ to a reasonable value (σ=0.25; see, e.g., ref. 80) and estimated $\beta_{A}$. The parameter $r_{X}$ represents the fraction of total personal contacts that individuals belonging to the X compartment have in the destination community (*SI Appendix*). We assume $r_{S}=0.5$ (i.e., each individual has, on average, half of the contacts in the place of work or study) and that $r_{E}=r_{P}=r_{A}=r_{R}=r_{S}$, while $r_{I}=r_{Q}=r_{H}=0$ (no extra province mobility of symptomatic infected, quarantined, and hospitalized individuals). Further assumptions aimed at reducing the number of parameters to be estimated are $\gamma_{Q}=\gamma_{I}=\gamma_{H},\gamma_{A}=2\gamma_{I}$, and $\alpha_{H}=\alpha_{I}$. We use information summarized in Table 1 to define prior distributions of key timescale parameters (Table 2). Moreover, the viral load of symptomatic cases is reportedly similar to that of the asymptomatic (81). We use such information to define the prior of the ratio $\beta_{I}/\beta_{A}$.

### Data Availability.

All data used in this manuscript are publicly available. COVID-19 epidemiological data for Italy are available at https://github.com/pcm-dpc/COVID-19. Mobility data at municipality scale are available at https://www.istat.it/it/archivio/139381. Population census data are available at http://dati.istat.it/Index.aspx?QueryId=18460.

## Supplementary Material

Supplementary File

Supplementary File

Supplementary File
