# Supplementary material for: Spread and dynamics of the COVID-19 epidemic in Italy: Effects of emergency containment measures
Source: Proc Natl Acad Sci U S A. 2020 Apr 23;117(19):10484–91. doi: 10.1073/pnas.2004978117 (PMC7229754; doi:10.1073/pnas.2004978117)
Supplement: Supplementary File [file pnas.2004978117.sapp.pdf]

Supporting Information
Spread and dynamics of the COVID-19 epidemic in Italy: effects of emergency containment measures

Marino Gatto[a,1], **Enrico Bertuzzo**[b], **Lorenzo Mari**[a], **Stefano Miccoli**[c], **Luca Carraro**[d,e], **Renato Casagrandi**[a], and **Andrea Rinaldo**[f,g,1]

[a]Dipartimento di di Elettronica, Informazione e Bioingegneria, Politecnico di Milano, 20133 Milano IT; [b]Dipartimento di Scienze Ambientali, Informatica e Statistica, Universitá Ca' Foscari Venezia, 30172 Venezia-Mestre IT; [c]Dipartimento di Meccanica, Politecnico di Milano, 20133 Milano IT; [d]Department of Aquatic Ecology, EAWAG, 8600 Dübendorf, CH; [e]Department of Evolutionary Biology and Environmental Studies, University of Zurich, 8057 Zurich, CH; [f]Laboratory of Ecohydrology, École Polytechnique Fédérale de Lausanne, 1015 Lausanne CH; [g]Dipartimento ICEA, Università di Padova, 35131 Padova IT

The Supporting Information (SI) is organized as follows. Section SI 1 reports details about the epidemiological data used in the modeling framework. Section SI 2 provides a review of the SEIR models used in the literature to describe the transmission of COVID-19. It also reports additional modeling details which complement and expand the methods as reported in the main document. A section SI 3 expands the results section of the main document with additional figures. The Section SI 4, describing Supporting Videos and their captions, closes then the SI.

SI 1. Data

A. Data. The present study is based exclusively on publicly available data. The main source is the *Dipartimento della Protezione Civile* (The Italian Civil Protection Department) GitHub repository (1), in which daily updates to the COVID-19 emergency are published. The database contains, starting from Feb. 24, 2020 and for each Italian *Regione* daily values for:

`ricoverati_con_sintomi` hospitalised patients with symptoms (not in ICU),

`terapia_intensiva` patients in ICU

`isolamento_domiciliare` patients in home confinement

`dimessi_guariti` cumulative number of recovered patients discharged from hospital

`deceduti` cumulative number of deaths

`totale_casi` cumulative number of confirmed cases

`tamponi` cumulative number of tests (*swabs*) performed.

Moreover, starting from Feb. 25, 2020, the cumulative number of confirmed cases is reported for each Italian *Provincia*. The cumulative number of hospitalized cases in each region is computed as the cumulative number of confirmed cases minus the cumulative number of patients in home confinement. To provide a proxy of the cumulative number of hospitalized cases at the province level, we downscaled the corresponding regional figure proportionally to the provincial cumulative number of cases of the corresponding day.

B. Geography of COVID-19 spread. The Supporting video 1 (Data animation) is an animation of the choropleth maps representing the spread of COVID-19 in Italy at the province level, spanning the time period from Feb. 25 (day 5) to Mar. 25 (day 34). The quantity represented is the ratio of the total number of confirmed cases to the resident population. The frames of the animation are collected in Fig. S3.

C. Containment measures.

- February 21, 2020: *Ordinanza del Ministero della Salute e Regione Lombardia* (Order of the Ministry of Health and Lombardy Region) (2). The municipalities of Codogno, Castiglione d'Adda, Casalpusterlengo, Fombio, Maleo, Somalglia, Bertonico, Terranova dei Passerini, Castelgerundo, and San Fiorano are recognized as the focus of COVID-19 cluster and strict social distancing measures enforced; public transportation is suspended.

- February 22, 2020: *Ordinanza del Ministero della salute e Regione Veneto* (Order of the Ministry of Health and Veneto Region) (3). The municipality of Vo' is subjected to analogous measures as in Lombardy for Codogno and nearby municipalities.

- February 23, 2020: *Decreto del Presidente del Consiglio dei Ministri 23 febbraio 2020* (Decree of the Prime Minister, Feb. 23, 2020) (4). Lockdown is enforced for the Codogno area in Lombardy and Vo' in Veneto, see Fig. S4.

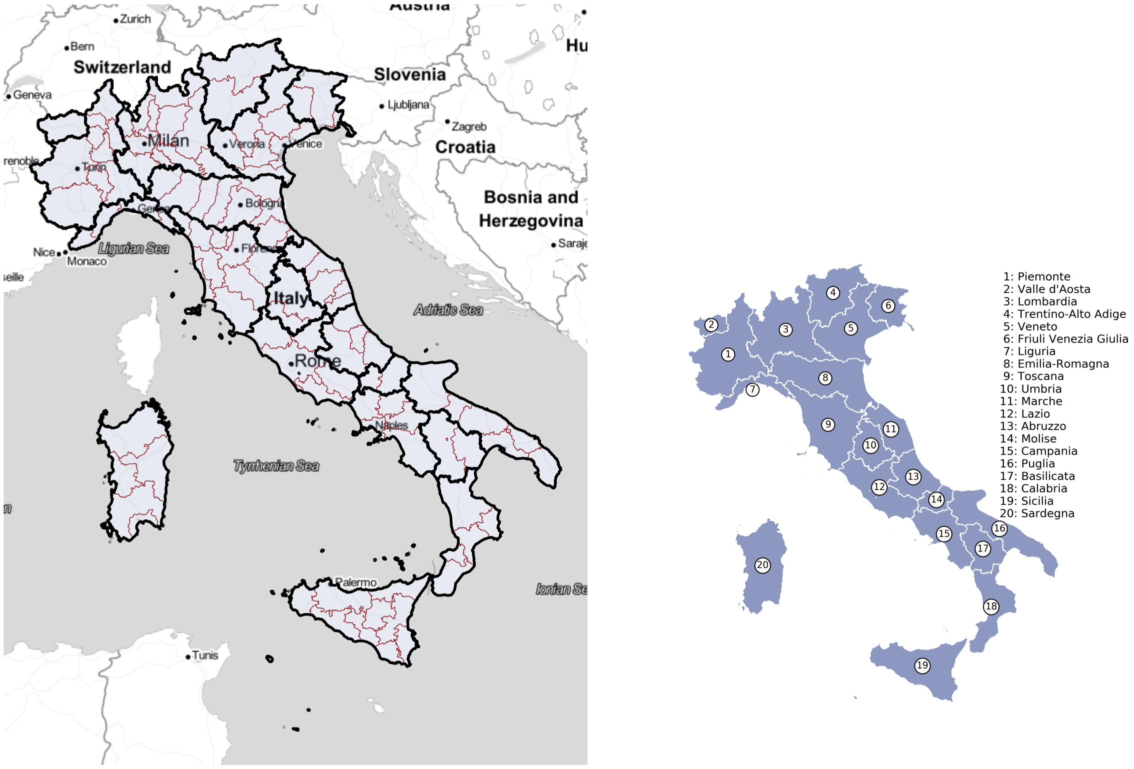

Map tiles copyright by Stamen Design, under CC BY 3.0. Data by OpenStreetMap, under ODbL.

Fig. S1. Italian administrative divisions: regions (thick black line) and provinces (thin brown line), with a legend of the regions names in Italian.

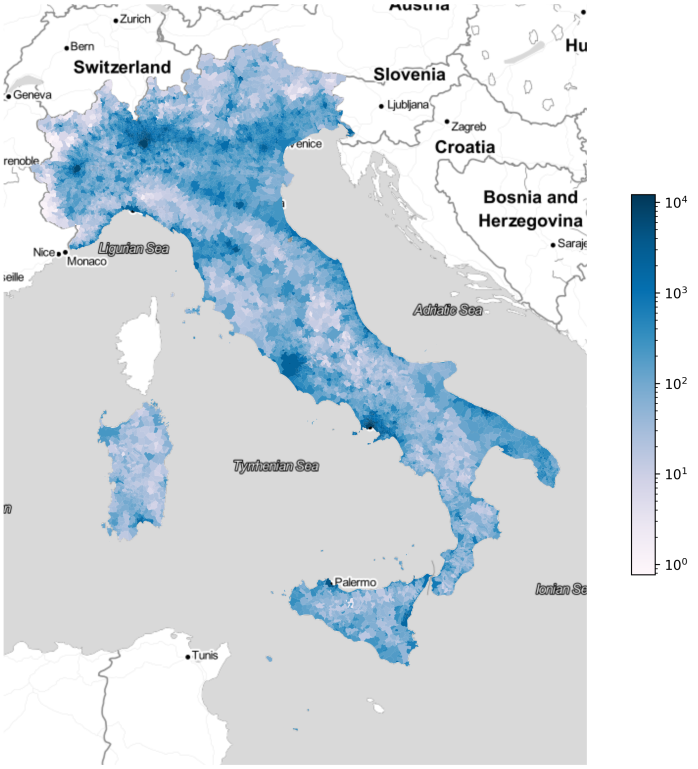

Map tiles copyright by Stamen Design, under CC BY 3.0. Data by OpenStreetMap, under ODbL.

Fig. S2. Population density $(1/\mathrm{km}^2)$ at the municipality level.

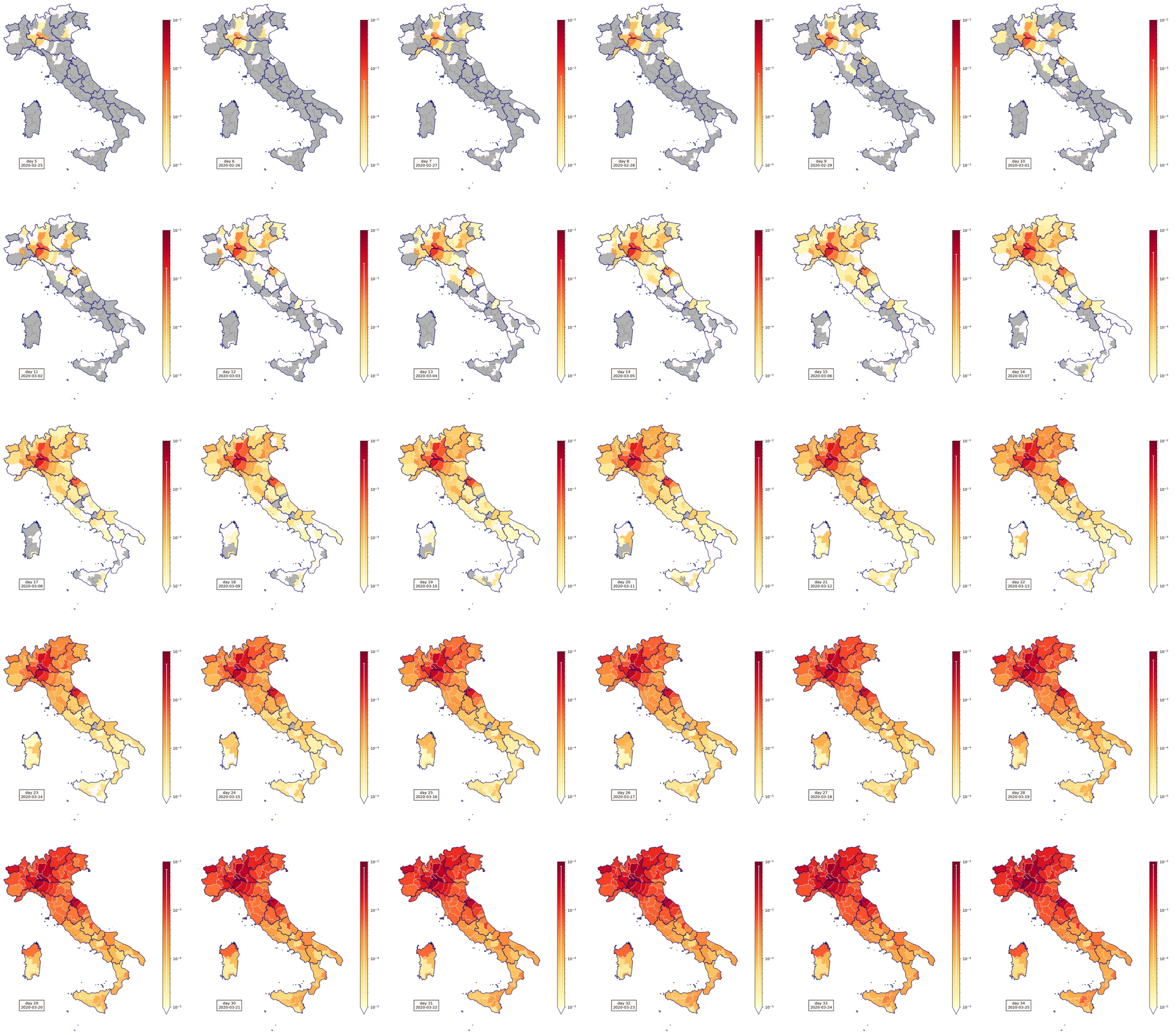

Fig. S3. Frames of `media/spatial_spread.mov`.

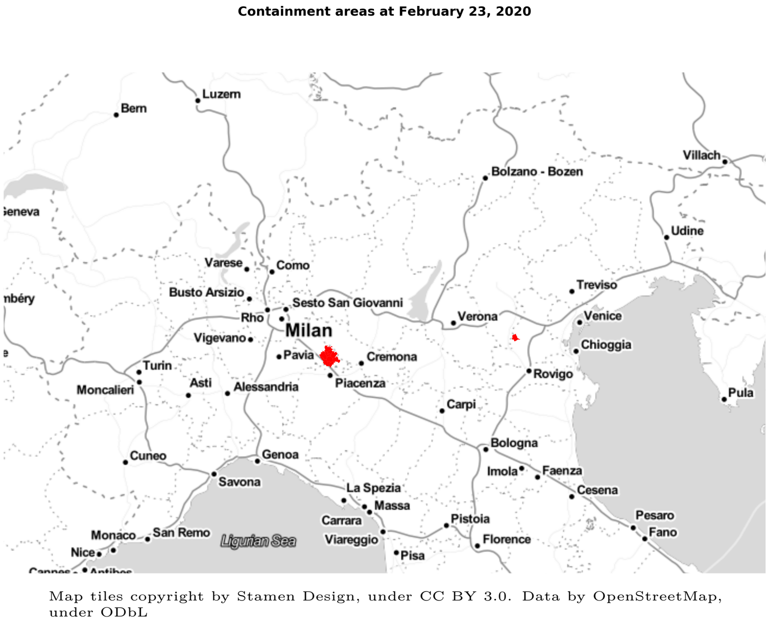

Fig. S4. Containment zones at Feb. 23, 2020 (4). Red areas are in lockdown.

- March 1, 2020: *Decreto del Presidente del Consiglio dei Ministri 1 marzo 2020*, (Decree of the Prime Minister, Mar. 1, 2020) (5). This decree confirms the strict lockdown for the Codogno area and Vo' ("red zone") and insitutes social distancing measures for the "orange zone" and "yellow zone", see Fig. S5.

- March 8, 2020: *Decreto del Presidente del Consiglio dei Ministri 8 marzo 2020* (Decree of the Prime Minister, Mar. 8, 2020) (6). Lockdown is enforced for Lombardy, and parts of Veneto, Piemonte, Marche; social distancing measures are extended to the whole nation, see Fig. S6.

- March 11, 2020: *Decreto del Presidente del Consiglio dei Ministri 11 marzo 2020* (Decree of the Prime Minister, Mar. 11) (7). Lockdown is extended to the whole nation. (6); see Fig. S7.

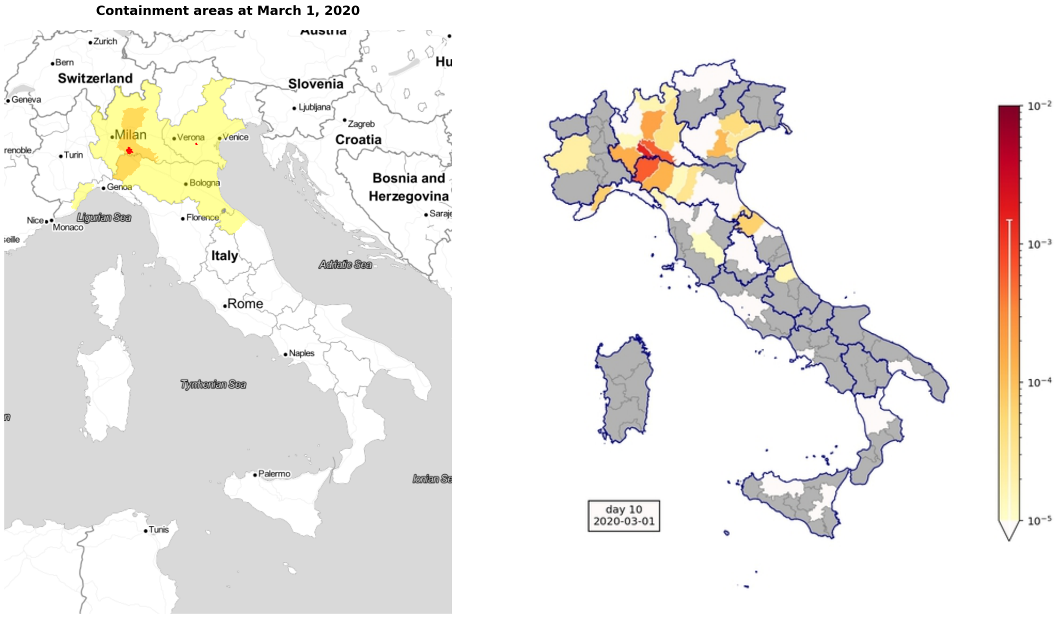

Map tiles copyright by Stamen Design, under CC BY 3.0. Data by OpenStreetMap, under ODbL

Fig. S5. a) Containment zones at Mar. 1, 2020 (4). Red areas are in strict lockdown; for yellow and orange areas social distancing measures apply. b) Distribution of confirmed COVID-19 cases, expressed as fraction over the resident population.

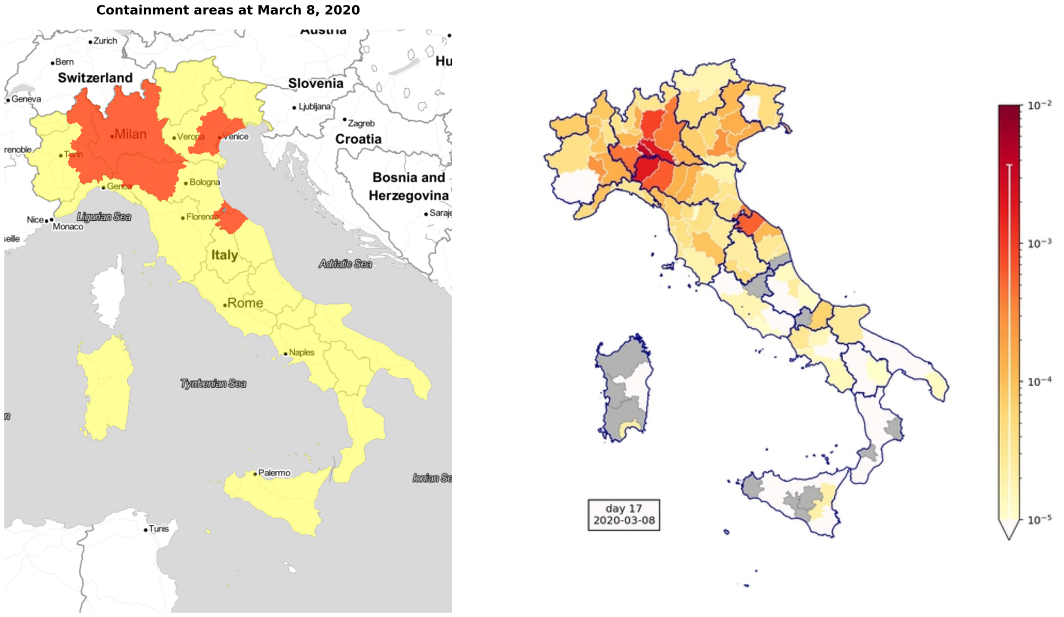

Map tiles copyright by Stamen Design, under CC BY 3.0. Data by OpenStreetMap, under ODbL.

Fig. S6. a) Containment zones at Mar. 8, 2020 (6). Red areas are in lockdown, for the whole nation social distancing is enforced. b) Distribution of confirmed COVID-19 cases, expressed as fraction over the resident population.

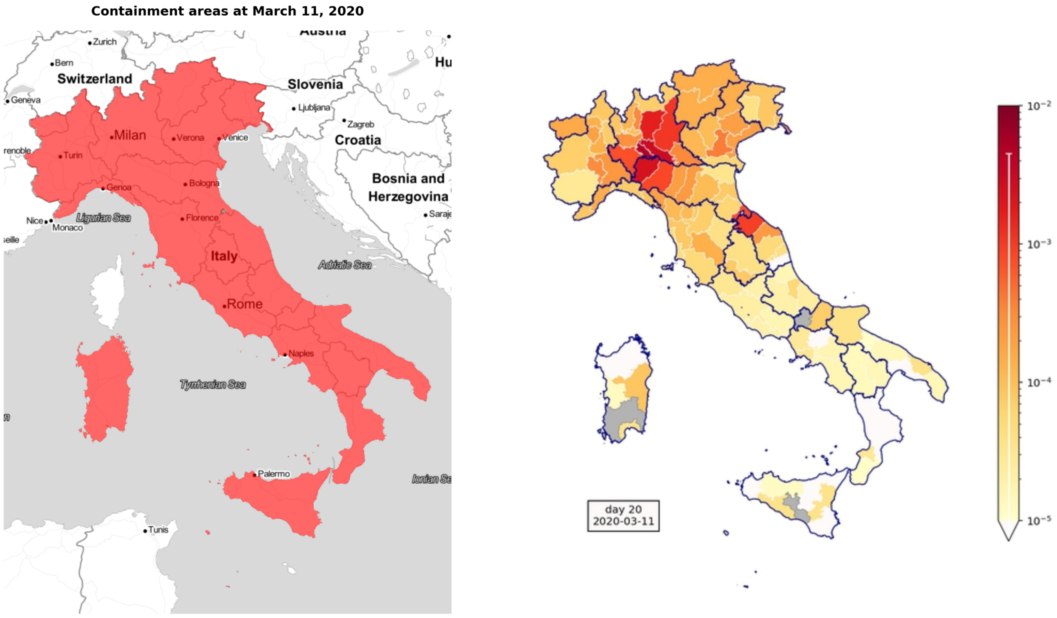

Map tiles copyright by Stamen Design, under CC BY 3.0. Data by OpenStreetMap, under ODbL.

Fig. S7. a) At Mar. 11, 2020 Italy is under lockdown (7). b) Distribution of confirmed COVID-19 cases, expressed as fraction over the resident population.

SI 2. model

A. Model.

Review of existing models. In the following we review different assumptions regarding the epidemiological compartments introduced in existing COVID-19 models:

- The exposed (E) remain in the compartment during the latency period; WHO (8) and (9) estimate the average latency period to be 7 days, (10–12) to be 5.2 days (95% CI 4.1-7.0) and (13) ranging between 3.44-3.69 days; these authors, however, often equate incubation to latency;

- there is evidence that the incubation period is longer than the latency period; however, some authors (11, 14, 15) assume the latency period to be equal to the incubation period, while others (10) assume that there is a lag of 5 days between the end of latency and the end of incubation or (12) assume first that the latency period is equal to the incubation period and then do a sensitivity analysis assuming that transmission could occur in the second half of the latency period. If latency and incubation do not coincide, it would be necessary to introduce a compartment (that we term P, namely post-latent) that lies in between the exposed and the symptomatic infectious (I);

- the infectious period begins with the end of the latency period and ends with the recovery (if recovery is meant to be the end of the infectious period, not the end of clinical signs) or with hospitalization/isolation; if we lump the post-latent together with the symptomatic into a unique compartment, we obtain the classical SEIR model (used for COVID-19, e.g., by (10, 14, 15)). If we term r the recovery rate and d the death rate, the average infectious period is $1/(r + d)$ for asymptomatic, non-hospitalized infectious. If all the diagnosed symptomatic people are hospitalized or quarantined at a rate h the average incubation period is $1/(r + d + h)$. As it is not always clear whether the estimate of the infectious period includes hospitalization/isolation, there might be wild variations in the estimates of the infectious period. For example, (14) estimate it to be 2.4 days (equal to the serial interval, estimated to be 8.4 days, minus the mean latent period, 6 days), (13) to be 3.5 days, (15) to be 2-8 days, with all these papers equating latency to incubation; (12) estimate it to be 2.9 days equating latency to incubation and assuming that all the infected are isolated sooner or later (so that they are no longer infectious). However, (12) estimate a delay of 6.1 days from onset to reporting. (9), instead, use a more complex SEIR model in which the infectious are divided into two sub-classes: the asymptomatic/mildly symptomatic (compartment A here) and the symptomatic (termed compartment I). They estimate the average infectious period to be 2.16 (range 1.64-3.10) days for the symptomatic infectious (who are isolated at a given rate) and 7.15 (range 4.81-13.93) days for asymptomatic infectious (who are not isolated). (16) do report values that include the stricter quarantine and isolation enforced in China.

- those papers (9, 13, 16) that make a distinction between symptomatic infectious (I) and asymptomatic (or undetected) infectious (A) report different fractions σ of people having symptoms or being detected among infected individuals: according to (9), who consider Wuhan only, it is 0.87 (SD=0.049), while (13), who consider the whole China, estimate that the fraction of reported infections is 0.14 (95% CI 0.10-0.18) during 10–23 January 2020, 0.65 (95% CI 0.60-0.69) during 24 January–3 February, 0.69 (95% CI 0.62-073) during 24 January–8 February.

A local model for COVID-19 transmission. To describe the transmission of COVID-19 in a well-mixed setting, we divide the population into epidemiological compartments corresponding to susceptible (S), exposed (E), post-latent (P), heavily symptomatic (I), asymptomatic/mildly symptomatic (A), hospitalized (H), quarantined (Q), recovered (R) and dead (D) individuals (Table S1). The dynamics of transmission is described by the following set of ordinary differential equations:

$$\begin{aligned}
\dot{S} &= -\lambda S \\
\dot{E} &= \lambda S - \delta_E E \\
\dot{P} &= \delta_E E - \delta_P P \\
\dot{I} &= \sigma \delta_P P - (\eta + \gamma_I + \alpha_I) I \\
\dot{A} &= (1-\sigma)\delta_P P - \gamma_A A \\
\dot{H} &= (1-\zeta)\eta I - (\gamma_H + \alpha_H) H \\
\dot{Q} &= \zeta \eta I - \gamma_Q Q \\
\dot{R} &= \gamma_I I + \gamma_A A + \gamma_H H \\
\dot{D} &= \alpha_I I + \alpha_H H \,.
\end{aligned} \tag{S1}$$

In the model, susceptible individuals become exposed to the viral agent upon contact with infectious individuals, assumed to be those in the post-latent, heavily symptomatic and asymptomatic/mildly symptomatic classes. Exposure occurs at a rate described by the frequency-dependent force of infection

$$\lambda = \frac{\beta_P P + \beta_I I + \beta_A A}{S + E + P + I + A + R}\,,$$

Variable	Definition
S	number of susceptible people
E	number of exposed people
P	number of post-latent infectious people
I	number of infectious people with severe symptoms
A	number of infectious people with no/mild symptoms
H	number of hospitalized people
Q	number of quarantined (home-isolated) people
R	number of recovered people
D	number of dead people

Table S1. State variables of the local COVID-19 transmission model.

Parameter	Definition
β_P	transmission rate of post-latent people
β_I	transmission rate of people with severe symptoms
β_A	transmission rate of people with no/mild symptoms
δ_E	latency rate
δ_P	post-latency rate
σ	fraction of infections with severe symptoms
η	removal rate of people with severe symptoms from the community
γ_I	recovery rate of people with severe symptoms
α_I	fatality rate of people with severe symptoms
γ_A	recovery rate of people with no/mild symptoms
ζ	fraction of severe infections being isolated at home
γ_H	recovery rate of hospitalized people
α_H	fatality rate of hospitalized people
γ_Q	recovery rate of quarantined (home-isolated) people

Table S2. Parameters of the local COVID-19 transmission model.

where β_P, β_I and β_A are the specific transmission rates of the three infectious classes. Exposed individuals are latently infected, i.e. not infectious, until they enter the post-latent stage (at rate δ_E) and become infectious. Post-latent individuals progress (at rate δ_P) to become either symptomatic infectious individuals who develop severe symptoms (with probability σ) or asymptomatic/mildly symptomatic individuals (with probability $1-\sigma$). Symptomatic infectious individuals exit this compartment if/when they are removed from the community (at rate η), recover from infection (at rate γ_I) or die (at rate α_I). Individuals removed from the community may be either hospitalized (with probability $1-\zeta$) or home-isolated (with probability ζ). Hospitalized patients may either recover from infection (at rate γ_H) or die because of it (at rate α_H). Asymptomatic/mildly symptomatic individuals, on the other hand, leave this compartment when they spontaneously recover from infection (at rate γ_A). All model parameters are summarized in Table S2.

The basic reproduction number. Close to the disease-free equilibrium (DFE) of model (S1), a state in which $S=N$ (with N being the total population size of the community) and $E=P=I=A=H=Q=R=D=0$, the Jacobian matrix of the infection subsystem $\{E,P,I,A\}$ is

$$\mathbf{J_0} = \begin{bmatrix} -\delta_E & \beta_P & \beta_I & \beta_A \\ \delta_E & -\delta_P & 0 & 0 \\ 0 & \sigma\delta_P & -(\eta+\alpha_I+\gamma_I) & 0 \\ 0 & (1-\sigma)\delta_P & 0 & -\gamma_A \end{bmatrix}.$$

Following (17), $\mathbf{J_0}$ can be decomposed into a transmission matrix

$$\mathbf{T} = \begin{bmatrix} 0 & \beta_P & \beta_I & \beta_A \\ 0 & 0 & 0 & 0 \\ 0 & 0 & 0 & 0 \\ 0 & 0 & 0 & 0 \end{bmatrix}$$

and a transition matrix

$$\mathbf{\Sigma} = \begin{bmatrix} -\delta_E & 0 & 0 & 0 \\ \delta_E & -\delta_P & 0 & 0 \\ 0 & \sigma\delta_P & -(\eta+\alpha_I+\gamma_I) & 0 \\ 0 & (1-\sigma)\delta_P & 0 & -\gamma_A \end{bmatrix},$$

so that $\mathbf{J_0} = \mathbf{T} + \mathbf{\Sigma}$. The next-generation matrix (NGM) with large domain, i.e. including all variables within the infection sub-system in addition to the states-at-infection, can then be found as

$$\mathbf{K_L} = -\mathbf{T}\mathbf{\Sigma}^{-1} = \begin{bmatrix} k_1 & k_2 & k_3 & k_4 \\ 0 & 0 & 0 & 0 \\ 0 & 0 & 0 & 0 \\ 0 & 0 & 0 & 0 \end{bmatrix} ,$$

with

$$k_1 = k_2 = \frac{\beta_P}{\delta_P} + \sigma \frac{\beta_I}{\eta + \alpha_I + \gamma_I} + (1-\sigma)\frac{\beta_A}{\gamma_A} , \quad k_3 = \frac{\beta_I}{\eta + \alpha_I + \gamma_I} \quad \text{and} \quad k_4 = \frac{\beta_A}{\gamma_A} .$$

The basic reproduction number is the spectral radius of the NGM with large domain (which coincides with the spectral radius of the NGM $\mathbf{K}$ including only the states-at-infection, namely E in the problem at hand), i.e.

$$\mathcal{R}_0 = \rho(\mathbf{K_L}) = k_1 = k_2 = \mathcal{R}_0^P + \mathcal{R}_0^I + \mathcal{R}_0^A ,$$

where

$$\mathcal{R}_0^P = \frac{\beta_P}{\delta_P} , \quad \mathcal{R}_0^I = \sigma \frac{\beta_I}{\eta + \alpha_I + \gamma_I} \quad \text{and} \quad \mathcal{R}_0^A = (1-\sigma)\frac{\beta_A}{\gamma_A}$$

represent the contributions of post-latent infectious people, infectious people with severe symptoms and infectious people with no/mild symptoms to the basic reproduction number, respectively.

The spatial spread of COVID-19. For a set of n connected communities, in each of which the abundances of susceptible individuals, exposed individuals, post-latent infectious individuals, infectious individuals with severe symptoms, infectious individuals with no/mild symptoms, hospitalized individuals, quarantined individuals, recovered individuals and dead individuals are S_i, E_i, P_i, I_i, A_i, H_i, Q_i, R_i and D_i, respectively, we have

$$\begin{aligned} \dot{S}_i &= -\lambda_i S_i \\ \dot{E}_i &= \lambda_i S_i - \delta_E E_i \\ \dot{P}_i &= \delta_E E_i - \delta_P P_i \\ \dot{I}_i &= \sigma \delta_P P_i - (\eta + \gamma_I + \alpha_I) I_i \\ \dot{A}_i &= (1-\sigma)\delta_P P_i - \gamma_A A_i \\ \dot{H}_i &= (1-\zeta)\eta I_i - (\gamma_H + \alpha_H) H_i \\ \dot{Q}_i &= \zeta \eta I_i - \gamma_Q Q_i \\ \dot{R}_i &= \gamma_I I_i + \gamma_A A_i + \gamma_H H_i \\ \dot{D}_i &= \alpha_I I_i + \alpha_H H_i , \end{aligned} \qquad \text{[S2]}$$

where the epidemiological parameters listed in Table S2 are assumed to be community-independent, while λ_i is the force of infection for community i. This term must account for contacts both within the local community and associated with mobility, say according to

$$\lambda_i = \sum_{j=1}^{n} C_{ij}^S \frac{\sum_{k=1}^{n} \left(\beta_P C_{kj}^P P_k + \beta_I C_{kj}^I I_k + \beta_A C_{kj}^A A_k \right)}{\sum_{k=1}^{n} \left(C_{kj}^S S_k + C_{kj}^E E_k + C_{kj}^P P_k + C_{kj}^I I_k + C_{kj}^A A_k + C_{kj}^R R_k \right)} ,$$

where C_{ij}^X (with $X \in \{S, E, P, I, A, R\}$) is the probability ($\sum_{j=1}^{n} C_{ij}^X = 1$ for all i's and X's) that individuals who belong to epidemiological compartment X and are from community i enter into contact with individuals who are present at community j as either residents or because they are traveling there from community k (note that i, j and k may coincide).

The basic reproduction number of the spatial model. Close to the DFE of model (S2), a state in which $S_i = N_i$ (with N_i being the total population size of community i) and $E_i = P_i = I_i = A_i = H_i = Q_i = R_i = D_i = 0$ for all i's, the Jacobian matrix of the infection subsystem $\{E_1, \ldots, E_n, P_1, \ldots, P_n, I_1, \ldots, I_n, A_1, \ldots, A_n\}$ reads

$$\mathbf{J_0^S} = \begin{bmatrix} -\delta_E \mathbf{I} & \mathbf{\Theta_P} & \mathbf{\Theta_I} & \mathbf{\Theta_A} \\ \delta_E \mathbf{I} & -\delta_P \mathbf{I} & \mathbf{0} & \mathbf{0} \\ \mathbf{0} & \sigma \delta_P \mathbf{I} & -(\eta + \alpha_I + \gamma_I)\mathbf{I} & \mathbf{0} \\ \mathbf{0} & (1-\sigma)\delta_P \mathbf{I} & \mathbf{0} & -\gamma_A \mathbf{I} \end{bmatrix} ,$$

where $\mathbf{I}$ and $\mathbf{0}$ are the identity and null matrices of size n, and the matrices $\mathbf{\Theta_P}$, $\mathbf{\Theta_I}$ and $\mathbf{\Theta_A}$ are defined as

$$\mathbf{\Theta_P} = \beta_P \mathbf{N}\mathbf{C_S}\mathbf{\Delta}^{-1}\mathbf{C_P}^T , \quad \mathbf{\Theta_I} = \beta_I \mathbf{N}\mathbf{C_S}\mathbf{\Delta}^{-1}\mathbf{C_I}^T \quad \text{and} \quad \mathbf{\Theta_A} = \beta_A \mathbf{N}\mathbf{C_S}\mathbf{\Delta}^{-1}\mathbf{C_A}^T ,$$

with $\mathbf{\Delta} = \text{diag}(\mathbf{u}\mathbf{N}\mathbf{C_S})$. In the previous expressions, $\mathbf{N}$ is a diagonal matrix whose nonzero elements are the population sizes N_i of the n communities, $\mathbf{u}$ is a unitary row vector of size n, and $\mathbf{C_X} = [C_{ij}^X]$ ($X \in \{S, P, I, A\}$) are row-stochastic matrices (i.e.

whose rows sum up to one) representing the spatially explicit contact probabilities. The spatial Jacobian can be decomposed into a spatial transmission matrix

$$\mathbf{T} = \begin{bmatrix} \mathbf{0} & \mathbf{\Theta_P} & \mathbf{\Theta_I} & \mathbf{\Theta_A} \\ \mathbf{0} & \mathbf{0} & \mathbf{0} & \mathbf{0} \\ \mathbf{0} & \mathbf{0} & \mathbf{0} & \mathbf{0} \\ \mathbf{0} & \mathbf{0} & \mathbf{0} & \mathbf{0} \end{bmatrix},$$

and a spatial transition matrix

$$\mathbf{\Sigma} = \begin{bmatrix} -\delta_E \mathbf{I} & \mathbf{0} & \mathbf{0} & \mathbf{0} \\ \delta_E \mathbf{I} & -\delta_P \mathbf{I} & \mathbf{0} & \mathbf{0} \\ \mathbf{0} & \sigma\delta_P \mathbf{I} & -(\eta + \alpha_I + \gamma_I)\mathbf{I} & \mathbf{0} \\ \mathbf{0} & (1-\sigma)\delta_P \mathbf{I} & \mathbf{0} & -\gamma_A \mathbf{I} \end{bmatrix},$$

so that $\mathbf{J_0} = \mathbf{T} + \mathbf{\Sigma}$. The NGM with large domain thus reads

$$\mathbf{K_L} = -\mathbf{T}(\mathbf{\Sigma})^{-1} = \begin{bmatrix} \mathbf{K_1} & \mathbf{K_2} & \mathbf{K_3} & \mathbf{K_4} \\ \mathbf{0} & \mathbf{0} & \mathbf{0} & \mathbf{0} \\ \mathbf{0} & \mathbf{0} & \mathbf{0} & \mathbf{0} \\ \mathbf{0} & \mathbf{0} & \mathbf{0} & \mathbf{0} \end{bmatrix},$$

with

$$\mathbf{K_1} = \mathbf{K_2} = \frac{1}{\delta_P}\mathbf{\Theta_P} + \sigma\frac{1}{\eta + \gamma_I + \alpha_I}\mathbf{\Theta_I} + (1-\sigma)\frac{1}{\gamma_A}\mathbf{\Theta_A}\,, \mathbf{K_3} = \frac{1}{\eta + \gamma_I + \alpha_I}\mathbf{\Theta_I} \quad \text{and} \quad \mathbf{K_4} = \frac{1}{\gamma_A}\mathbf{\Theta_A}\,.$$

The spatial NGM $\mathbf{K}$ accounting only for the states-at-infection (E_i) is simply $\mathbf{K_1}$, and the basic reproduction number can be found as:

$$\mathcal{R}_0 = \rho(\mathbf{K_L}) = \rho(\mathbf{K}) = \rho(\mathbf{G_P} + \mathbf{G_I} + \mathbf{G_A})\,,$$

where

$$\mathbf{G_P} = \frac{\beta_P}{\delta_P}\mathbf{N}\mathbf{C_S}\mathbf{\Delta}^{-1}\mathbf{C_P}^T\,, \quad \mathbf{G_I} = \frac{\beta_I}{\eta + \gamma_I + \alpha_I}\mathbf{N}\mathbf{C_S}\mathbf{\Delta}^{-1}\mathbf{C_I}^T \quad \text{and} \quad \mathbf{G_A} = \frac{\beta_A}{\gamma_A}\mathbf{N}\mathbf{C_S}\mathbf{\Delta}^{-1}\mathbf{C_A}^T$$

are three spatially explicit generation matrices describing the contributions of post-latent infectious people, infectious people with severe symptoms and infectious people with no/mild symptoms to the production of new infections close to the DFE.

B. Application to the spread of COVID-19 in Italy. The model is run at the scale of second-level administrative divisions, i.e. provinces and metropolitan cities. In addition to the epidemiological parameters, assumed to be spatially homogeneous for the sake of simplicity, the model requires georeferenced, nation-wide information about population distribution and mobility. Official data about resident population at various spatial resolutions is provided yearly (last update: January 1st, 2019) by the Italian National Institute of Statistics (Istituto Nazionale di Statistica, ISTAT; data available at http://dati.istat.it/Index.aspx?QueryId=18460). On the other hand, the latest official country-wide assessment of mobility for Italy has been conducted in the context of the 2011 national census. The results of the assessment, reflecting commuting fluxes, are distributed in the form of origin-destination matrices at the scale of municipalities (third-level divisions; data available online at https://www.istat.it/it/archivio/139381). Municipality-level mobility fluxes are upscaled to the provincial level using the administrative divisions of 2019 (which are slightly different compared to those of 2011).

To check whether the 2011 mobility fluxes are still informative of the current ones, we have compared them with an origin-destination matrix provided by Lombardia region for the regional fluxes of year 2020. This matrix (available online at https://www.dati.lombardia.it/Mobilit-e-trasporti/Matrice-OD2020-Passeggeri/hyqr-mpe2) is actually the result of a statistical projection four years ahead of mobility data collected in 2016. Although the comparison between the mobility fluxes provided by ISTAT in 2011 and those estimated by Lombardia region for 2020 shows a growing transport demand (evaluated in terms of the sheer number of people traveling between provinces; Figure S10), the spatial patterns of human movement (evaluated as the fraction of people traveling between provinces; Figure S11) seem to be remarkably preserved over a 10-year-long time interval. Although we cannot exclude that this result could be limited to Lombardia region only, it is at least indicative of the fact that mobility patterns inferred from the 2011 country-wide census may still be relevant for the purpose of this work.

For each second-level administrative unit, say i, two quantities are extracted from the 2011 ISTAT data, namely the fraction p_i of mobile people, i.e. the residents of i who declared themselves as commuters, and the fraction q_{ij} of mobile people between i and all other administrative units $j = 1 \dots n$ (including $j = i$). The contact probabilities C_{ij}^X ($X \in \{S, E, P, I, A, R\}$) are then defined based on the quantities p_i and q_{ij}. Specifically, we assume

$$C_{ij}^X = \begin{cases} (1-p_i) + (1-r_X)p_i + r_X p_i q_{ij} & \text{if } i = j \\ r_X p_i q_{ij} & \text{otherwise}\,, \end{cases}$$

where r_X ($0 \le r_X \le 1$) is an additional parameter describing the fraction of contacts occurring while individuals in epidemiological compartment X are traveling. In other words, for community i, the social contacts of non-mobile people (a fraction $1 - p_i$ of the community size), those of mobile people that do not occur during travel (a fraction $1 - r_X$ of total contacts for people in epidemiological compartment X) and those associated with mobility for people who travel within their

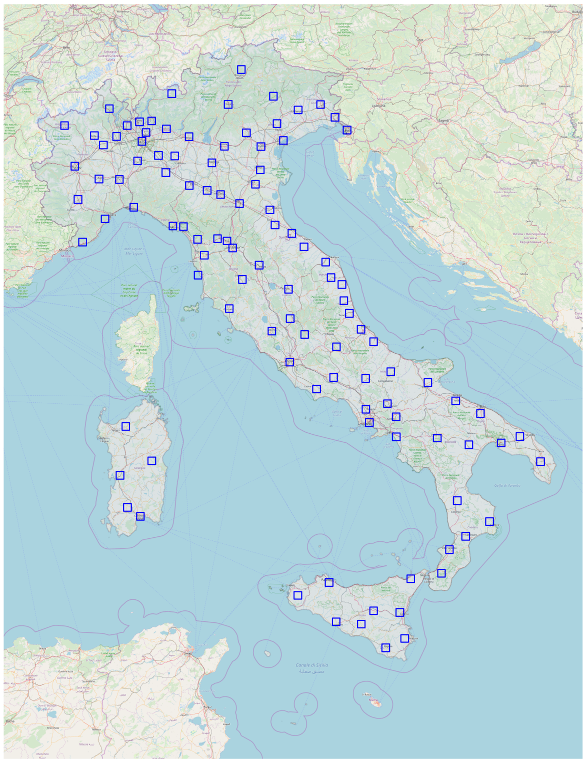

Fig. S8. Grid at province level

community (a fraction q_{ii} of mobile people), contribute to social mixing within the community. Conversely, the contacts occurring between two different communities, say i and j, are a fraction r_X of the total contacts of the individuals in epidemiological compartment X, multiplied by the probability p_i that people from i travel (independently of the destination) and the probability q_{ij} that the travel occurs between i and j.

As the duration of the simulated outbreak (around a month) is comparable to the average times spent by the individuals in the various model compartments, it is important to correctly describe the distribution of residence times. The formulation described in equations S2 implies exponentially-distributed residence times in each compartment. However, the distribution of crucial stages, like the latent and the hospitalization periods, are reportedly hump-shaped with a positive mode, and are typically modeled with a gamma distribution (see e.g. (11)). To properly describe gamma-distributed residence times, we split the exposed E and hospitalized H compartments into two and three sequential, identical compartments, respectively, as customary (see e.g (12) for COVID-19). Since this assumption involves only state variables that either describe a pure temporal transition (exposed individuals, for whom no mortality processes are accounted for) or do not contribute to transmission (hospitalized people, who are assumed not to contribute to the spread of infection), the expression of $\mathcal{R}_0$ remains unchanged (18).

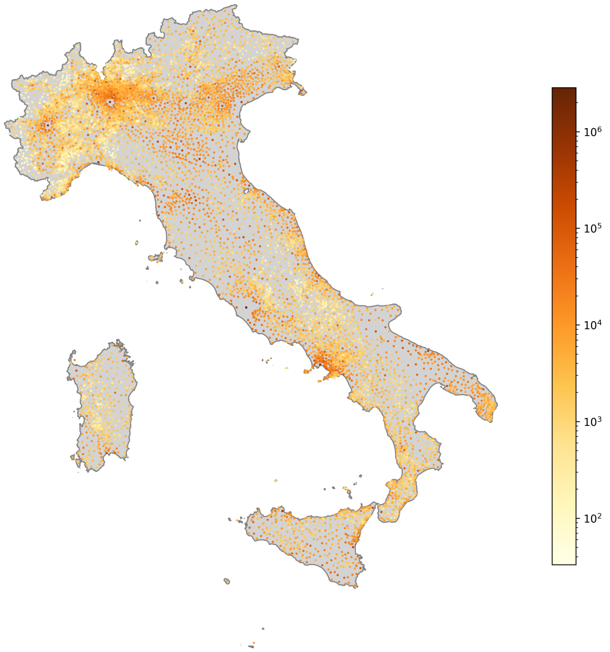

Fig. S9. Grid at municipality level. The nodes are color-coded for the resident population.

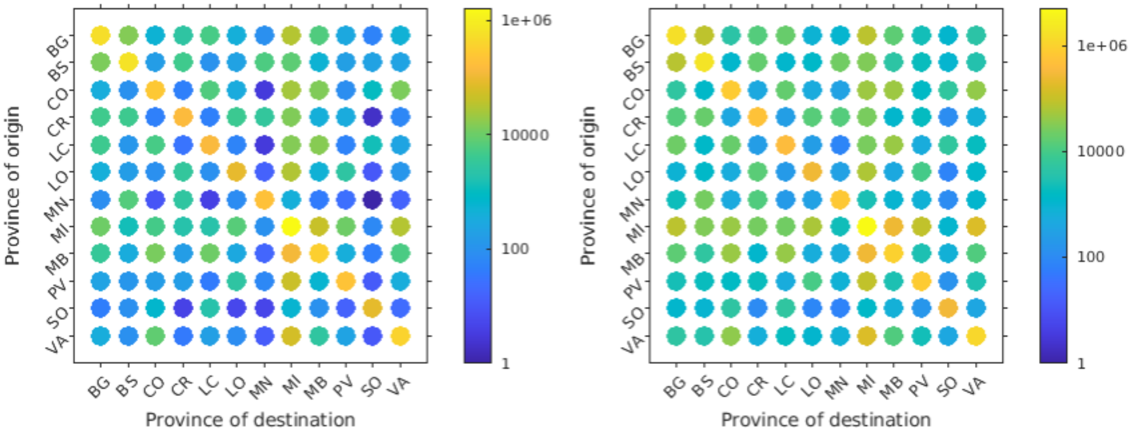

Fig. S10. Comparison between the mobility fluxes (number of travelers) among the 12 provinces of Lombardia region estimated according to the 2011 national census (left) or a projection for year 2020 made by Lombardia region based on 2016 data (right).

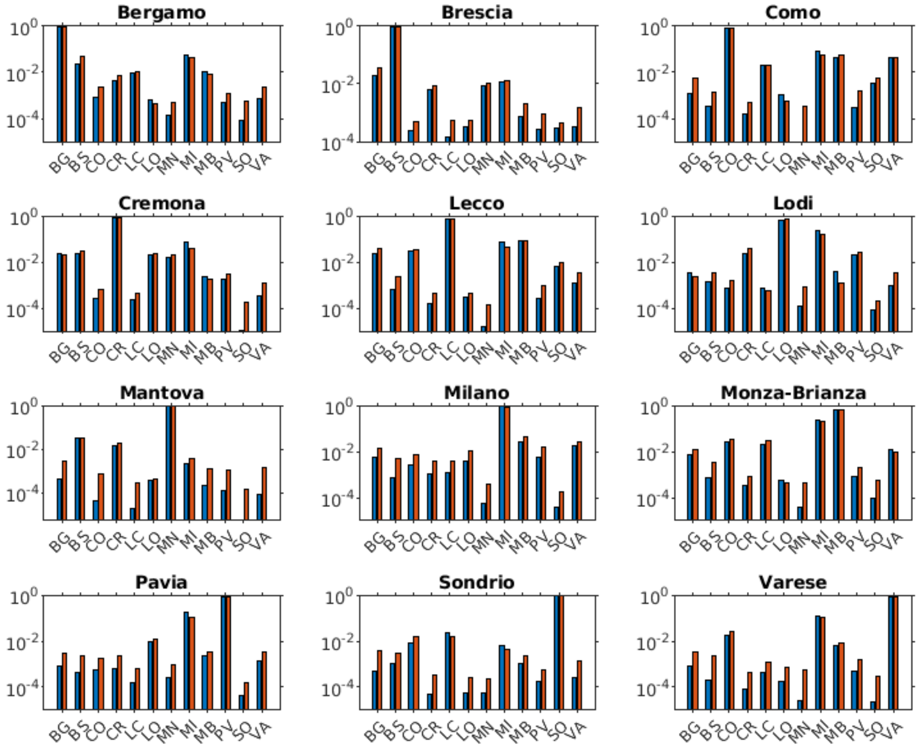

Fig. S11. Comparison between the outgoing mobility patterns (fraction of mobile people traveling from one province, top labels, to all others, horizontal axis label) among the 12 provinces of Lombardia region estimated according to the 2011 national census (blue bars) or a projection for year 2020 made by Lombardia region based on 2016 data (red bars).

SI 3. Supplementary results

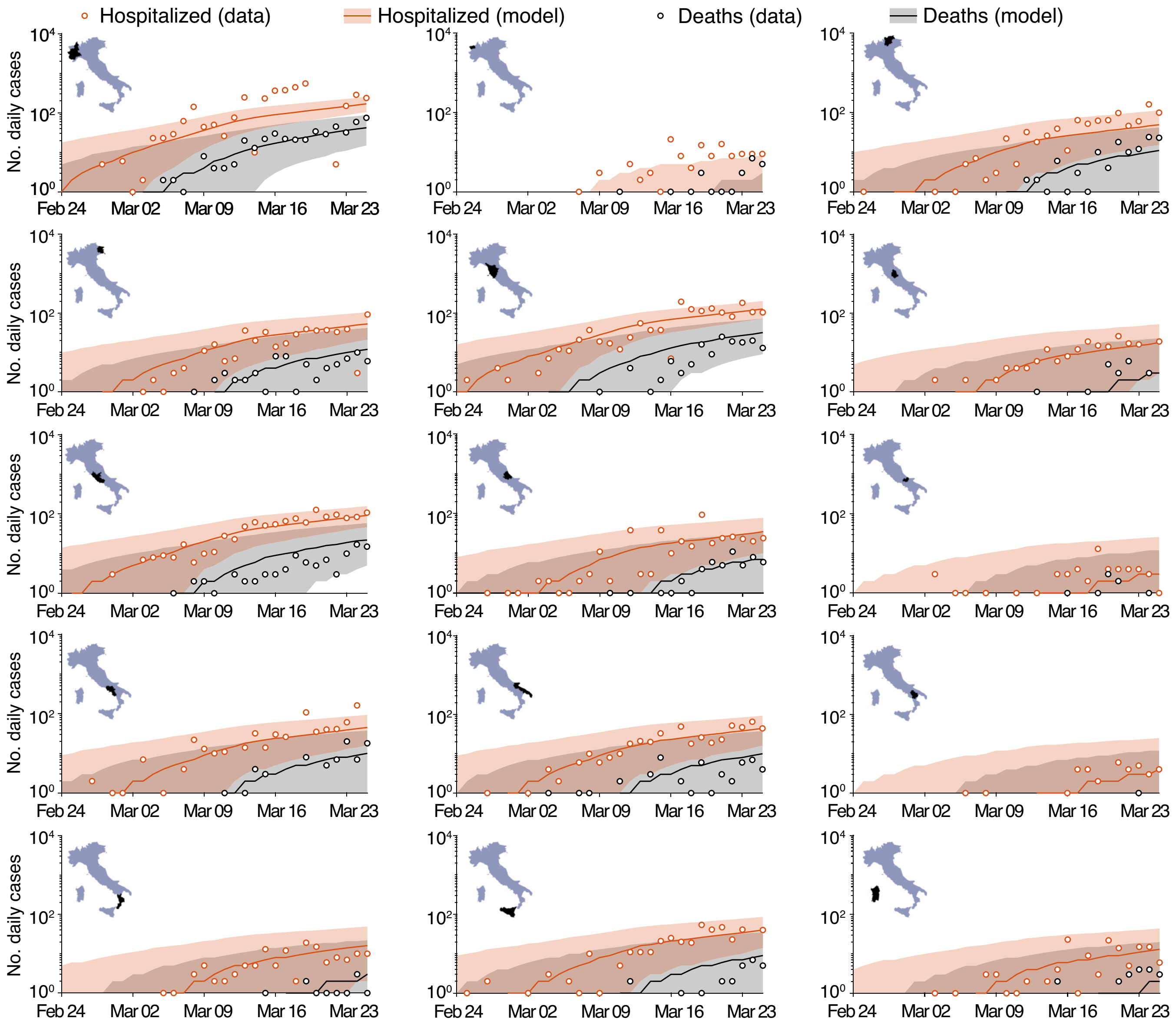

Fig. S12. Reported and simulated aggregate cases for COVID-19 spread in Italy for the 15 regions not reported in Figure 3 of the main text. Shaded areas identify 95% confidence intervals.

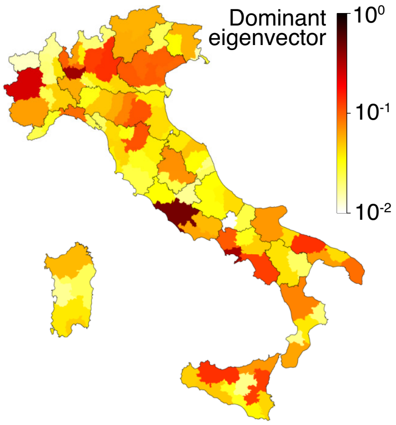

Fig. S13. Dominant eigenvector of the spatial Jacobian matrix (exposed components). The eigenvector corresponding to the leading eigenvalue of the Jacobian represents the expected spatial distribution of cases in the asymptotic phase of exponential epidemic growth (19). Parameter values as in Table 2 in the main text.

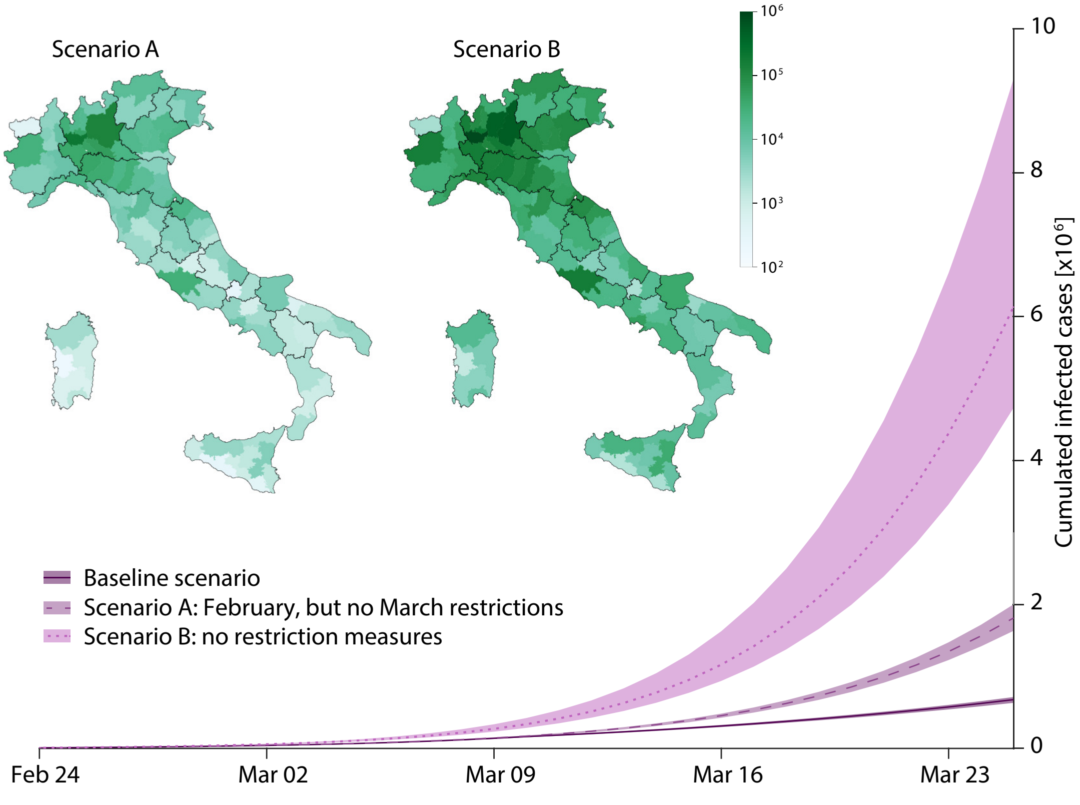

Fig. S14. Scenarios of averted infections as a result of the sequence of progressive mobility restrictions and social distancing in Italy. The three curves represent the analog of Figure 4 of the main text, only computed on cumulative infections (the integral of the flux λS). Symbols as in Figure 4 of the main text. As of March 25, the median number of averted infections due to the implementation of all restriction measures is $6.49 \cdot 10^6$ [95% CI: $4.81 - 10.05 \cdot 10^6$]. Note that quantiles of averted cases (hospitalizations in Figure 4 of the main text, and infections in this Figure) were calculated by resampling the differences between predicted distributions of cases without and with measures. As a result, the so-obtained median averted cases differ with respect to the differences of the medians of averted cases without and with measures.

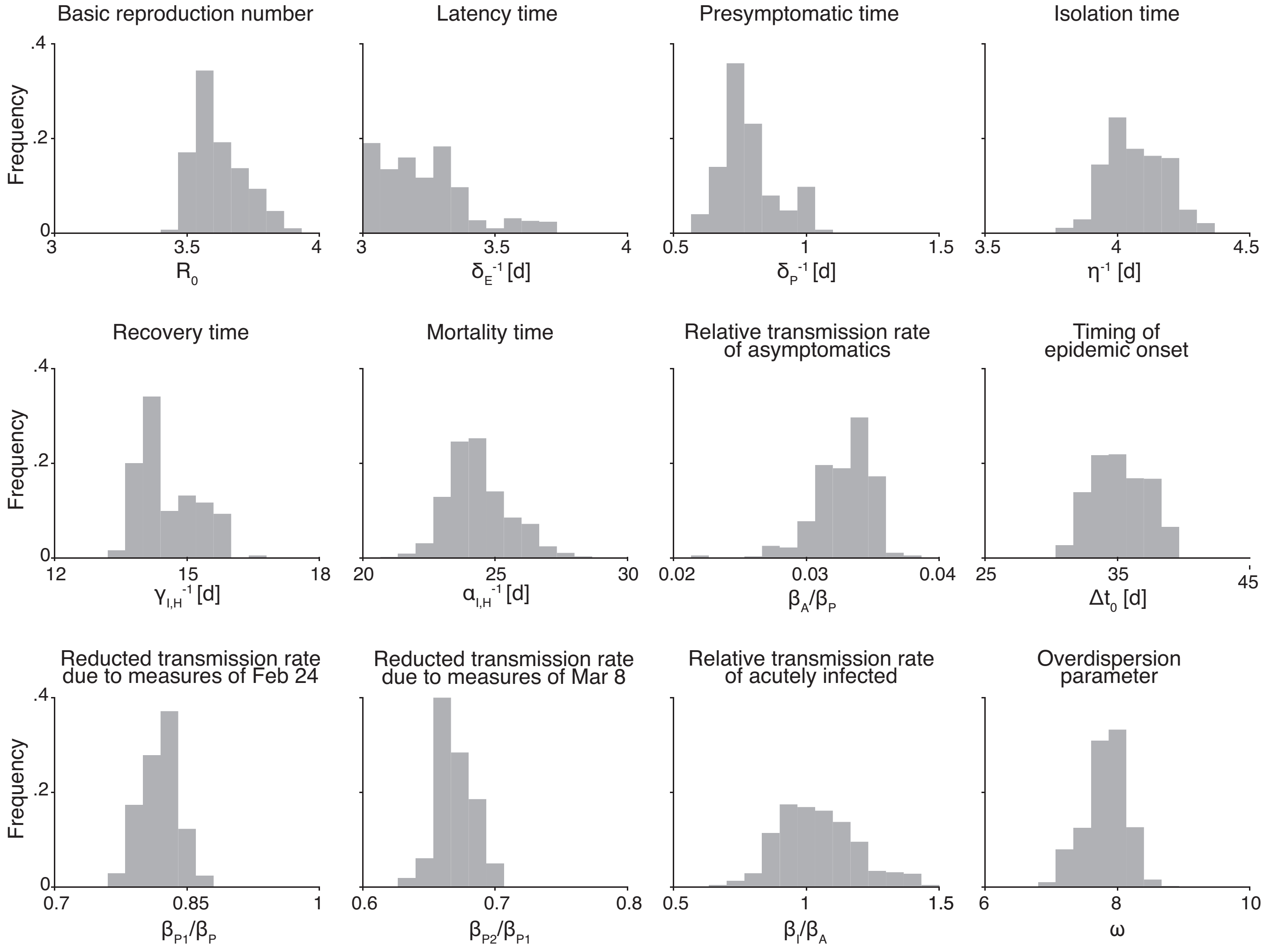

Fig. S15. Posterior distributions for the estimated parameters (see Table 2 in the main text).

SI 4. Supporting Videos

The Supporting Video 1 (`Data animation`) is an animation of the choropleth maps representing the spread of COVID-19 in Italy at the province level, spanning the time period from Feb. 25 (day 5) to Mar. 25 (day 34). The quantity represented is the ratio of the total number of confirmed cases to the resident population.

The Supporting Video 2 (`Model and Data animation`) is an animation showing the comparison between simulated and recorded spatio-temporal evolution of the cumulative number of severe cases that required hospitalization. Left panel: model simulation at the municipality scale (N=7926) using the median estimated parameters reported in Table 2 of the main text. Central Panel: simulation at the province scale (N=107) used to estimate model parameters. Right panel: recorded data at the province scale. Circle size is proportional to the logarithm of the cumulative cases. Top inset shows the corresponding cumulative number of severe cases for the whole Italy: circles represent data, shaded area the model simulation.

References

1. Dipartimento della Protezione Civile (2020) COVID-19 Italia - monitoraggio situazione. https://github.com/pcm-dpc/COVID-19 (Accessed: 25 March 2020).
2. Ministero della Salute (2020) Ordinanza del Ministero della Salute e Regione Lombardia. http://www.trovanorme.salute.gov.it/norme/renderNormsanPdf?anno=2020&codLeg=73314&parte=1%20&serie=null.
3. Ministero della Salute (2020) Ordinanza del Ministero della Salute e Regione Veneto. http://www.trovanorme.salute.gov.it/norme/renderNormsanPdf?anno=2020&codLeg=73320&parte=1%20&serie=null.
4. Presidente del Consiglio dei Ministri (2020) Decreto del presidente del consiglio dei ministri 23 febbraio 2020. https://www.gazzettaufficiale.it/eli/id/2020/02/23/20A01228/sg.
5. Presidente del Consiglio dei Ministri (2020) Decreto del presidente del consiglio dei ministri 1 marzo 2020. https://www.gazzettaufficiale.it/eli/id/2020/03/01/20A01381/sg.
6. Presidente del Consiglio dei Ministri (2020) Decreto del presidente del consiglio dei ministri 8 marzo 2020. https://www.gazzettaufficiale.it/eli/id/2020/03/08/20A01522/sg.
7. Presidente del Consiglio dei Ministri (2020) Decreto del presidente del consiglio dei ministri 11 marzo 2020. https://www.gazzettaufficiale.it/eli/id/2020/03/11/20A01605/sg.
8. World Health Organization (2020) Coronavirus disease (COVID-2019) situation reports. https://www.who.int/emergencies/diseases/novel-coronavirus-2019/situation-reports/ (Accessed: 25 March 2020).
9. Tang B, et al. (2020) Estimation of the transmission risk of the 2019-nCoV and its implication for public health interventions. *J. Clinical Med.* 9(2):462.
10. Wang H, et al. (2020) Phase-adjusted estimation of the number of coronavirus disease 2019 cases in Wuhan, China. *Cell Discovery* 6(1).
11. Li Q, et al. (2020) Early transmission dynamics in Wuhan, China, of Novel Coronavirus–infected pneumonia. *N. Engl. J. Med.* 382(13):1199–1207.
12. Kucharski AJ, et al. (2020) Early dynamics of transmission and control of COVID-19: a mathematical modelling study. *The Lancet Inf. Dis.*
13. Li R, et al. (2020) Substantial undocumented infection facilitates the rapid dissemination of novel coronavirus (SARS-CoV2). *Science* p. eabb3221.
14. Wu JT, Leung K, Leung GM (2020) Nowcasting and forecasting the potential domestic and international spread of the 2019-nCoV outbreak originating in Wuhan, China: a modelling study. *The Lancet* 395(10225):689–697.
15. Chinazzi M, et al. (2020) The effect of travel restrictions on the spread of the 2019 novel coronavirus (COVID-19) outbreak. *Science* p. eaba9757.
16. Tang B, et al. (2020) An updated estimation of the risk of transmission of the novel coronavirus (2019-nCoV). *Infectious Disease Modelling* 5:248–255.
17. Diekmann O, Heesterbeek J, Roberts M (2010) The construction of next-generation matrices for compartmental epidemic models. *J. Roy. Soc. Interface* 7(47):873–885.
18. Krylova O, Earn DJD (2013) Effects of the infectious period distribution on predicted transitions in childhood disease dynamics. *Journal of The Royal Society Interface* 10(84):20130098.
19. Gatto M, et al. (2012) Generalized reproduction numbers and the prediction of patterns in waterborne disease. *Proc. Natl. Acad. Sci. USA* 48:19703–19708.
