# Supplementary figures and images for: Spread and dynamics of the COVID-19 epidemic in Italy: Effects of emergency containment measures
Source: Proc Natl Acad Sci U S A. 2020 Apr 23;117(19):10484–91. doi: 10.1073/pnas.2004978117 (PMC7229754; doi:10.1073/pnas.2004978117)

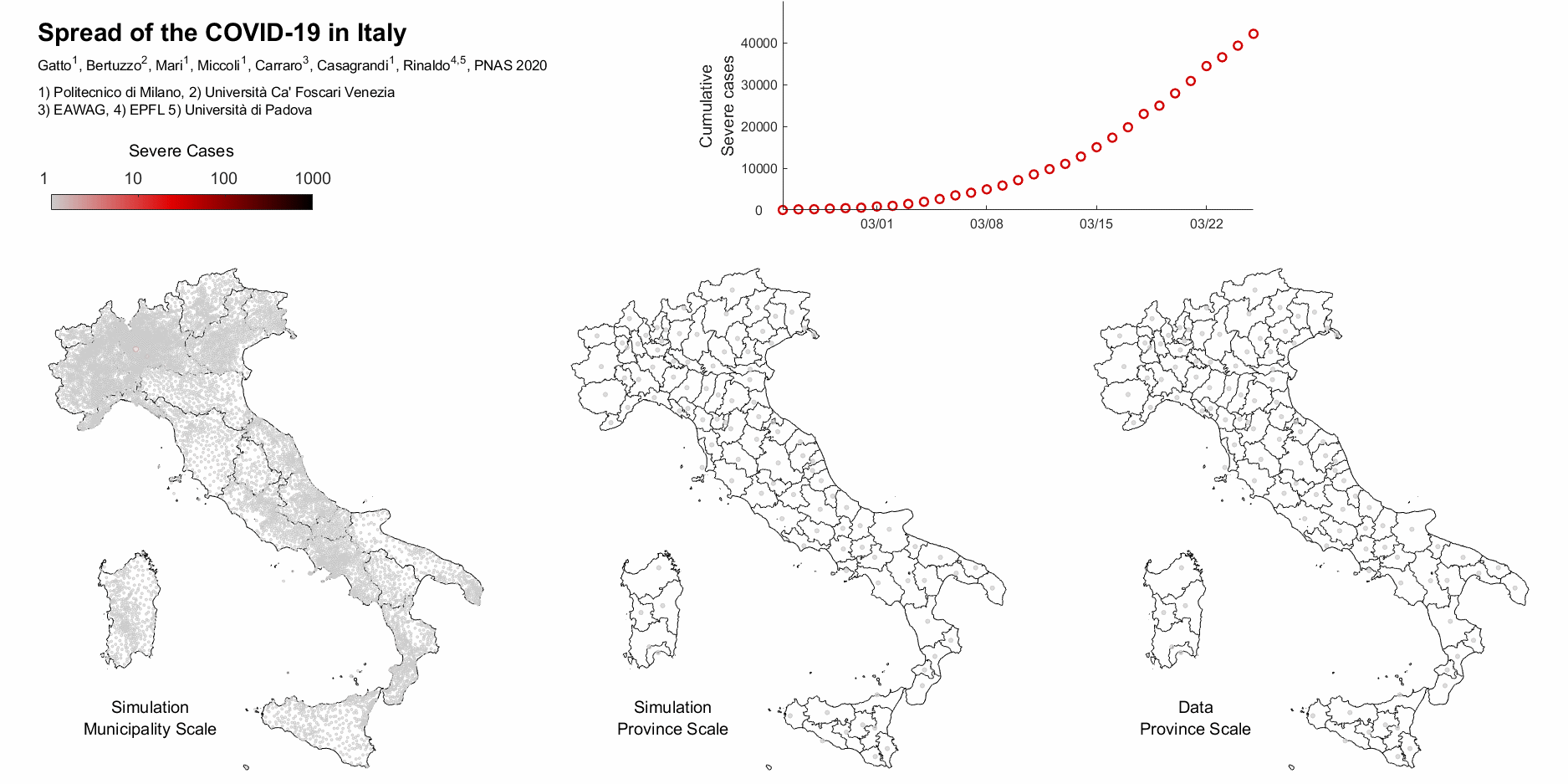

Supplement: Supplementary File [file pnas.2004978117.sm02.gif]
